# Restoring Tactile Sensation Using a Triboelectric Nanogenerator

**DOI:** 10.1021/acsnano.0c10141

**Published:** 2021-06-17

**Authors:** Iftach Shlomy, Shay Divald, Keshet Tadmor, Yael Leichtmann-Bardoogo, Amir Arami, Ben M. Maoz

**Affiliations:** † Department of Biomedical Engineering, Tel Aviv University, Tel Aviv, 69978, Israel; ‡ Sagol School of Neuroscience, Tel Aviv University, Tel Aviv, 69978, Israel; § Hand Surgery Department, Microsurgery and Peripheral Nerve Surgery Unit, Sheba Medical Center, Tel Hashomer, 52621, Israel; ∥ The Center for Nanoscience and Nanotechnology, Tel Aviv University, Tel Aviv, 69978, Israel; ⊥ Sackler School of Medicine, Tel Aviv University, Tel Aviv, 69978, Israel

**Keywords:** tactile restoration, peripheral nerve injury, triboelectric effect nano generator, TENG, implanted biosensor

## Abstract

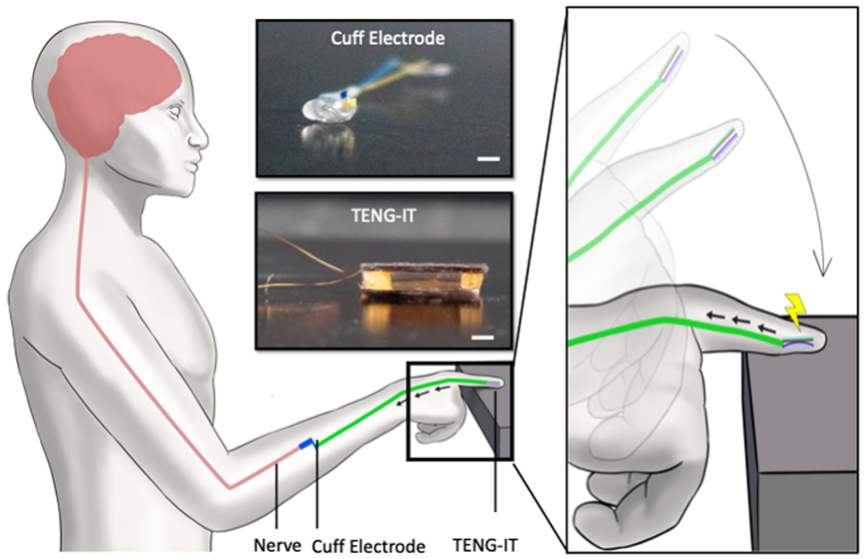

Loss of tactile sensation is a common occurrence in patients with traumatic peripheral nerve injury or soft tissue loss, but as yet, solutions for restoring such sensation are limited. Implanted neuro-prosthetics are a promising direction for tactile sensory restoration, but available technologies have substantial shortcomings, including complexity of use and of production and the need for an external power supply. In this work, we propose, fabricate, and demonstrate the use of a triboelectric nanogenerator (TENG) as a relatively simple, self-powered, biocompatible, sensitive, and flexible device for restoring tactile sensation. This integrated tactile TENG (TENG-IT) device is implanted under the skin and translates tactile pressure into electrical potential, which it relays via cuff electrodes to healthy sensory nerves, thereby stimulating them, to mimic tactile sensation. We show that the device elicits electrical activity in sensory neurons *in vitro*, and that the extent of this activity is dependent on the level of tactile pressure applied to the device. We subsequently demonstrate the TENG-IT *in vivo*, showing that it provides tactile sensation capabilities (as measured by a von Frey test) to rats in which sensation in the hindfoot was blocked through transection of the distal tibial nerve. These findings point to the substantial potential of self-powered TENG-based implanted devices as a means of restoring tactile sensation.

Traumatic peripheral nerve injury (TPNI) is a common disorder that affects 2.8% of trauma patients, and can result in lifelong disability,^1^ chronic pain, and diminished quality of life.^2,3^ A common effect of TPNI is a loss of tactile sensation,^4,5^ which not only interferes with patients’ daily lives but also increases susceptibility to injury. Currently, few solutions are available for restoration of tactile sensation. The gold standard solution is surgical nerve reconstruction by nerve autograft, nerve conduits,^6^ or nerve allografts.^7,8^ Unfortunately, nerve reconstruction can only be performed in a limited time period (*i.e*., in the first two years after injury), and it requires healthy skin with viable end-organs. Moreover, even when these conditions are met, its success rate is low.^9^

An alternative promising avenue for the restoration of tactile sensation is the development of wearable or implanted neuro-prosthetic devices that simulate the experience of touch. This simulation is achieved by translating pressure cues around the damaged area into electrical signals that can subsequently be processed by the brain. Several such devices have been proposed and implemented, using various technologies, and innovations are continually emerging in the field^10−16^ (see Table S1 for a summary of the properties of the main tools). Technologies that have received particular attention include computer–brain interfaces^14−16^ and “electronic skin” that mimics not only the sensory properties of skin but also some of its biological properties (*e.g*., stretchability).^11^ Neuro-prosthetic technologies are still in their infancy, however, and only a few have undergone proof-of-principle testing *in vivo.*(10) Moreover, the tools developed thus far have several key shortcomings: First, they are expensive and complex to implement, with some (*e.g*., electronic skin) requiring supplementary support platforms.^12^ These features suggest that the process of adapting current technologies into devices that are appropriate for widespread clinical use is likely to be prolonged, and, without significant advances in cost reduction, the finished product may still be inaccessible to many patients. Second, current neuro-prosthetic technologies require a power source—typically an external power source or a battery. Such power sources may be inconvenient to replace, and they might risk introducing toxic materials in the case of malfunction.^13^ Third, the neuro-prosthetic technologies that have been implemented in patients tend to require long periods of training and adjustment.^17^

Clearly, there is much room for development in the design of implanted devices aimed at restoring tactile sensation. In addition to overcoming the shortcomings of current and emerging technologies, such a device should ideally fulfill several criteria. First, the device should be made of biocompatible material, to prevent damage to the tissue surrounding the implant.^18^ In addition, the device should be flexible, durable, and small. Another crucial feature is a wide range of sensitivity, corresponding to normal human pressure perception,^19^ which ranges from a few kPa for a gentle touch to tens of kPa for object manipulation. An additional desirable feature is simplicity of design and implantation, toward enhancing the accessibility of the device to the general population of patients.

Herein, we propose leveraging a recently developed technology to create a class of practical tactile restoration devices that can fulfill the criteria outlined above, while overcoming the shortcomings of existing neuro-prosthetic solutions. The technology at the focus of our work is the triboelectric nanogenerator (TENG),^20−23^ which converts mechanical energy into electricity by a conjunction of triboelectrification and electrostatic induction^24^ (Figure 1a). Several recent studies have demonstrated the great potential of TENG technology in diverse applications (*e.g*., evaluation of water quality, harvesting blue energy),^25−27^ and specifically in the biomedical industry.^22,28−32^ The latter studies have shown that the TENG can be used to harvest biomechanical energy and can thereby facilitate nerve repair or serve as an autonomous power source for medical devices.^22,28^ Crucially, the TENG’s sensitivity to pressure has been suggested to make it a promising candidate for tactile sensory applications.^29^ In this work, we begin to realize this potential, by creating an integrated tactile (IT) sensory restoration device (TENG-IT), and demonstrating its functionality *in vivo*.

**Figure 1:**
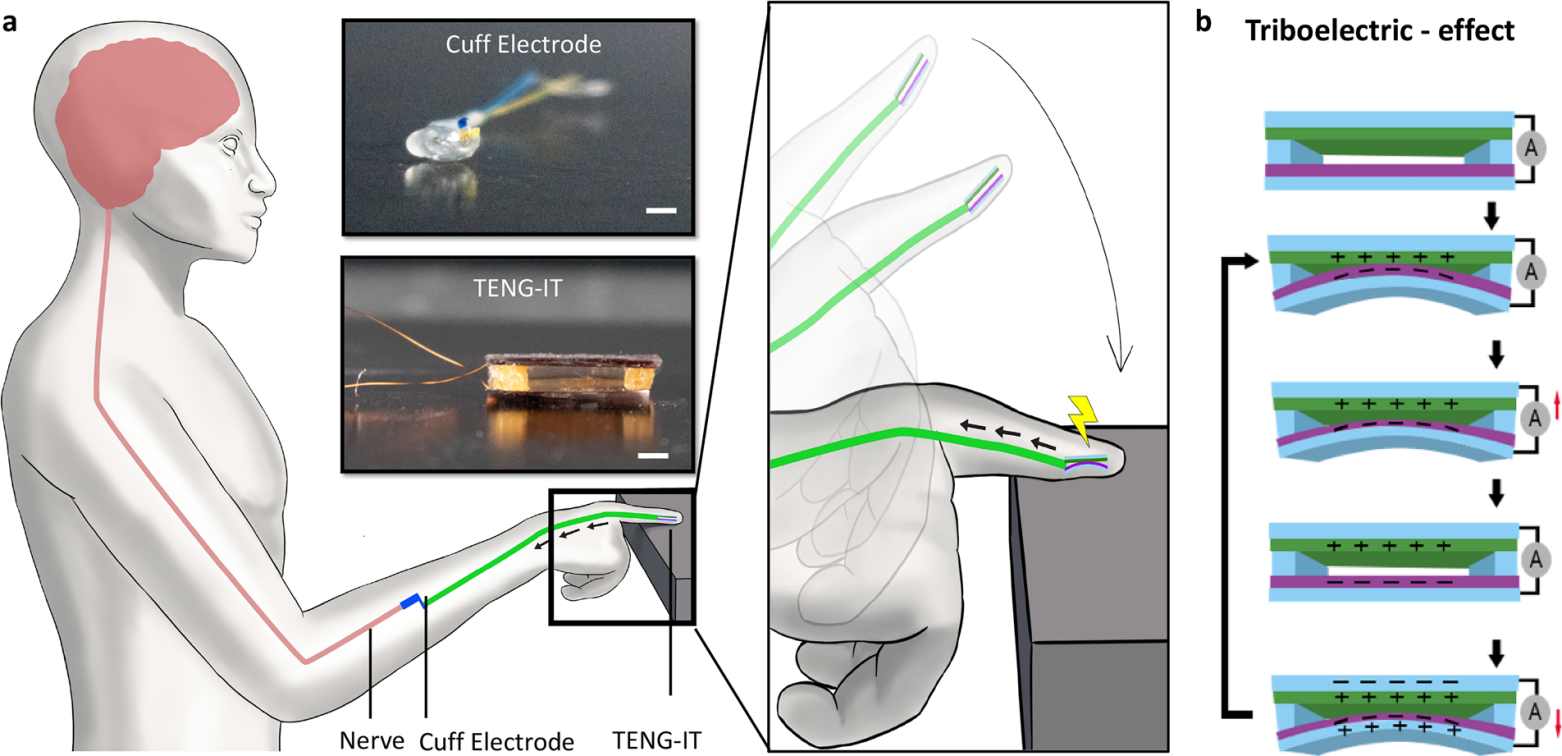
Illustration of the TENG-IT. a. Use of TENG-IT for restoring tactile sensation. The TENG-IT is implanted under the skin (*e.g*., of a desensitized finger). Upon application of tactile pressure to the device, the TENG-IT generates an electrical signal, which is delivered using isolated cables to a stimulating cuff electrode wrapped around the closest undamaged afferent nerve fiber, which transduces a touch sensation signal to the CNS. Upper inset: top: Photo of a cuff electrode (scale bar: 1 mm); bottom: Photo of a TENG-IT device (scale bar: 1 mm; a larger version than the device used in our experiments is pictured for clarity of presentation). b. Schematics of the TENG, which converts mechanical energy into electricity using the triboelectric effect.

The TENG-IT is a self-powered device that is implanted under the skin (*e.g*., at the fingertip) and that transforms touch into voltage, which is transduced to healthy sensory nerves via cuff electrodes, to excite peripheral neurons proportionally to the pressure that is applied on the device (Figure 1b). The device comprises a small number of components and is constructed from affordable materials; moreover, its fabrication process is straightforward. In what follows, we elaborate on the design of the TENG-IT device and demonstrate its ability to excite peripheral neurons *in vitro* and to provide tactile sensory capabilities to rodents in which a segment of a sensory nerve was removed. Overall, this work provides an affordable, accessible, self-powered, and sensitive device for restoring tactile sensation.

## Results and Discussion

### TENG-IT Development and Characterization

#### Selection of Materials and Evaluation of Capacity to Generate Electrical Potential

**Figure 2:**
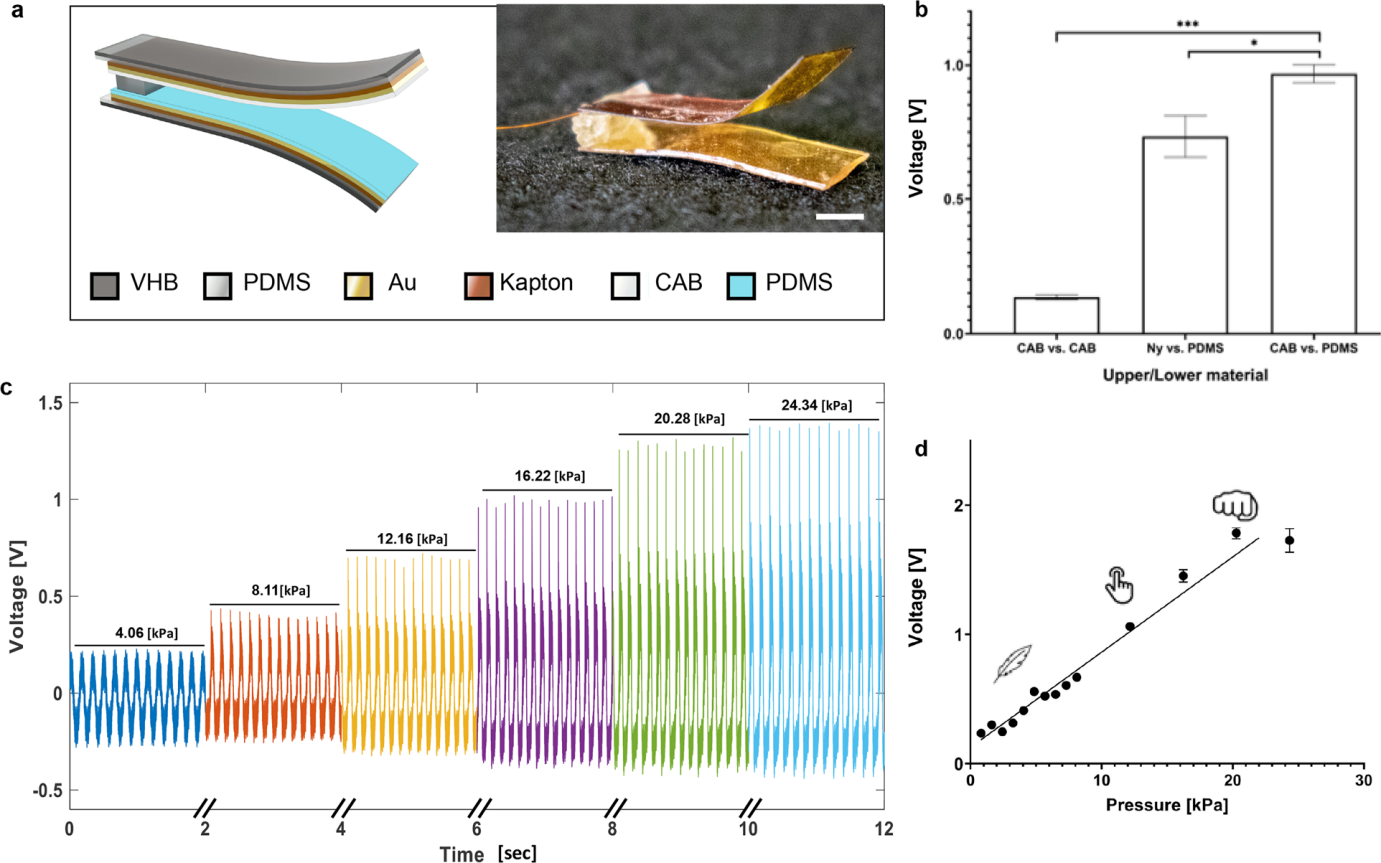
TENG-IT characterization. a. Schematic of the TENG-IT layers (left) and a photo of the TENG-IT (right) (scale bar: 2 mm). b. Mean peak-to-peak electrical output modulation of TENG-IT (5 mm × 5 mm), as a function of the materials used for the friction layers. c. Output performance of TENG-IT (5 mm × 5 mm) for different levels of pressure applied. d. TENG-IT (5 mm × 5 mm) response to physiological pressure. We observe a linear correlation (*R*^2^ = 0.97) between the average peak-to-peak output voltage and pressure applied to the device.

Although the TENG was introduced only 8 years ago,^33^ many TENGs have since been developed, and extensive research has been devoted to identifying the best materials and design for such nanogenerators.^24,34^ Building on this research, we identified and tested several materials that would enable us to create a device that is stable, sensitive to pressure along the physiological range, durable, biocompatible, and capable of generating a large triboelectric effect (Figure 2a,b). We note that the materials selected for the TENG, as well as the fabrication process, are aligned with established practices outside the biomedical domain;^25−27^ Our device demonstrates the possibility of implementation of a TENG for restoration of tactile sensory capabilities (an application that has been suggested but not yet realized^29^) and specifically, the use of cuff electrodes to relay TENG-generated potential to healthy nerves (as elaborated in subsequent sections).

In general, a TENG is composed of positive and negative dielectric materials, which are positioned on top of the metal, which serves as an electrode (Figure 1a, Figure 2a, Figure S1; see Materials and Methods section for details). In this work, polydimethylsiloxane (PDMS) was chosen as the negatively charged dielectric material, and both nylon (Ny) and cellulose acetate butyrate (CAB) were tested as the positively charged layer, as they are known to be biocompatible and flexible, and they have the potential to generate large electrical potential. For the metal (the electrode), we used a thin layer of gold, which was evaporated on Kapton for stability (in our initial experiments, the Kapton layer was 125 μm thick).

After constructing devices (surface area: 25 mm^2^ [5 mm × 5 mm]) from the candidate material combinations (Ny:PDMS, CAB:PDMS), in addition to a “control” device constructed from a single, positively charged dielectric material (CAB:CAB), we evaluated each device’s capacity to generate electric potential in response to touch (SI Movie 1). As expected, the control device produced a negligible change in voltage in response to touch, whereas devices constructed from combinations of positive and negative dielectric materials produced significant changes in voltage (Figure 2b). CAB:PDMS produced higher output voltage (0.97 ± 0.03 V) than did Ny:PDMS (0.73 ± 0.08 V); moreover, it was more convenient to work with and more stable (the Ny electrodes peeled off after a short usage period; Figure S2). Accordingly, the CAB:PDMS combination was used in all subsequent experiments.

#### Response to Physiological Pressure

Our next step in characterizing and evaluating the TENG-IT was to verify that the device operates as expected in response to pressure within the physiological range^19^—and specifically, that the relationship between the output voltage and the pressure applied fits the following equation:^35^
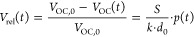
1where *V*_rel_ is the relative change in voltage, *V*_OC_ is the TENG-IT output voltage at a specific time point, *d*_0_ is the maximal distance (*i.e*., the size of the gap) between the two dielectric layers, *S* is the surface area of the TENG-IT (see Materials and Methods section and Figure S3), *k* represents the TENG-IT’s elasticity, and *p* is the pressure applied. As can be seen in Figure 2c,d, the TENG-IT operates in the physiological range of pressure (as low as 1 kPa and high as 20 kPa), and the voltage–pressure relationship has high linear correlation to eq 1 (*R*^2^ = 0.97).

Next, we examined whether the sensitivity of the TENG was dependent on the thickness of the Kapton layer supporting the electrode. To do so, we fabricated two different TENGs, one containing a “thick” (125 μm) layer of Kapton, and the other containing a “thin” (13 μm) layer. We then tested each device’s electrical output. As Figure S4 demonstrates, the device that was fabricated using the “thin” layer of Kapton was more sensitive than the device with the “thick” layer of Kapton, and produced higher voltage for a given amount of pressure. Though the “thick” layer of Kapton was used in the final version of the device—as this layer provided adequate sensitivity for our proof of principle—subsequent iterations of the TENG-IT might incorporate alternative thicknesses of the Kapton layer to achieve optimal sensitivity.

#### Durability

**Figure 3:**
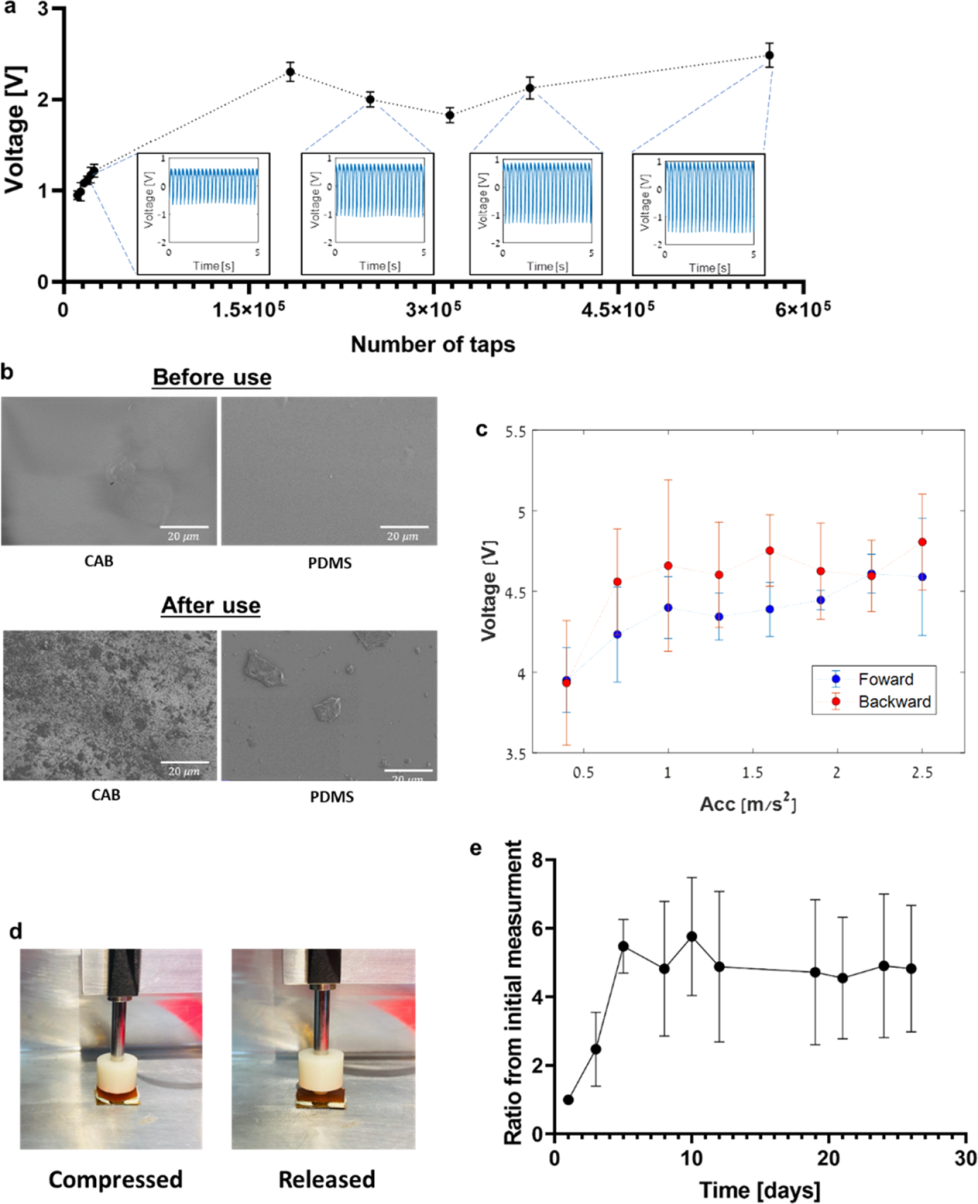
a. Average peak-to-peak output voltage from TENG-IT. Insets in blue show raw data corresponding to 5 s intervals after specific time points. Pressure applied was 15 kPa at 4.5 Hz. b. SEM images of the TENG-IT (5 mm × 5 mm): top view of each layer of dielectric material (CAB or PDMS). The roughness of the surface of the dielectric materials changes for both PDMS and CAB, after >0.5 million taps of 15 kPa. c. Average peak-to-peak output voltage of the TENG-IT in response to a set of increasing and decreasing accelerations (pressures) (labeled “forward” for increasing pressures [blue] and “backward” for decreasing pressures [red] that immediately followed; *n* = 3). d. Example of TENG-IT compression. e. Average peak-to-peak output voltage divided by the initial peak-to-peak measurement (*n* = 3). The pressure was applied by a motor moving in a sinusoidal motion at 1 Hz at peak acceleration of 3.95 m/s^2^.

To evaluate the durability of the device, we applied repeated pressure of 15 kPa at a frequency of 4.5 Hz over 36 h, resulting in more than 580,000 strong “finger taps” on the device (Figure 3a). Interestingly, over the course of the first 11,000 taps, the output voltage from the TENG increased to 128% of its starting value. After approximately 170,000 taps, the final output voltage arrived at saturation (2.487 ± 0.133 V), corresponding to 261% of the initial value (Figure 3a). In an effort to identify the cause of this increase in voltage over time, we examined scanning electron microscope (SEM) images of the surface area of the dielectric materials; the images had been recorded over the course of the experiment (Figure 3b). We observed that the surface of the dielectric materials (mainly the CAB) became rougher over time. As a TENG’s triboelectric effect is highly dependent on its surface area^36^ (eq 1), the roughening of the surface apparently increased the output voltage for a given pressure (Figure 3a,b).

Next, we evaluated the robustness of the TENG-IT in the presence of multiple forces over time (Figure 3c,d). During force application, we verified that the two layers touched each other and went back to their original state (Figure 3b). The results show that the dynamic range of the TENG-IT is not significantly affected by the application of high pressure, which is a crucial condition for *in vivo* use.

#### Resistance to Biological Conditions

Exposure to biological conditions such as moisture, body temperature, and salinity might cause degradation via corrosion, swelling, and hydrolysis. To evaluate how the TENG-IT might respond to such exposure over time, and to validate the long-term stability of the device, the following procedure was performed. Over a period of 26 days, TENG-IT devices were kept in PBS solution in an incubator at 37 °C—an environment simulating biological conditions. The device’s output voltage in response to peak acceleration of 3.95 m/s^2^ was measured 3 times a week for 30 min. We observed that after a stabilization period of ∼5 days, the output voltage remained relatively stable as compared to the initial measurement (Figure 3e). Notably, this pattern resembles the pattern observed in our initial durability test, in the absence of biological conditions (Figure 3a).

### TENG-IT Activates Sensory Neurons in Vitro

**Figure 4:**
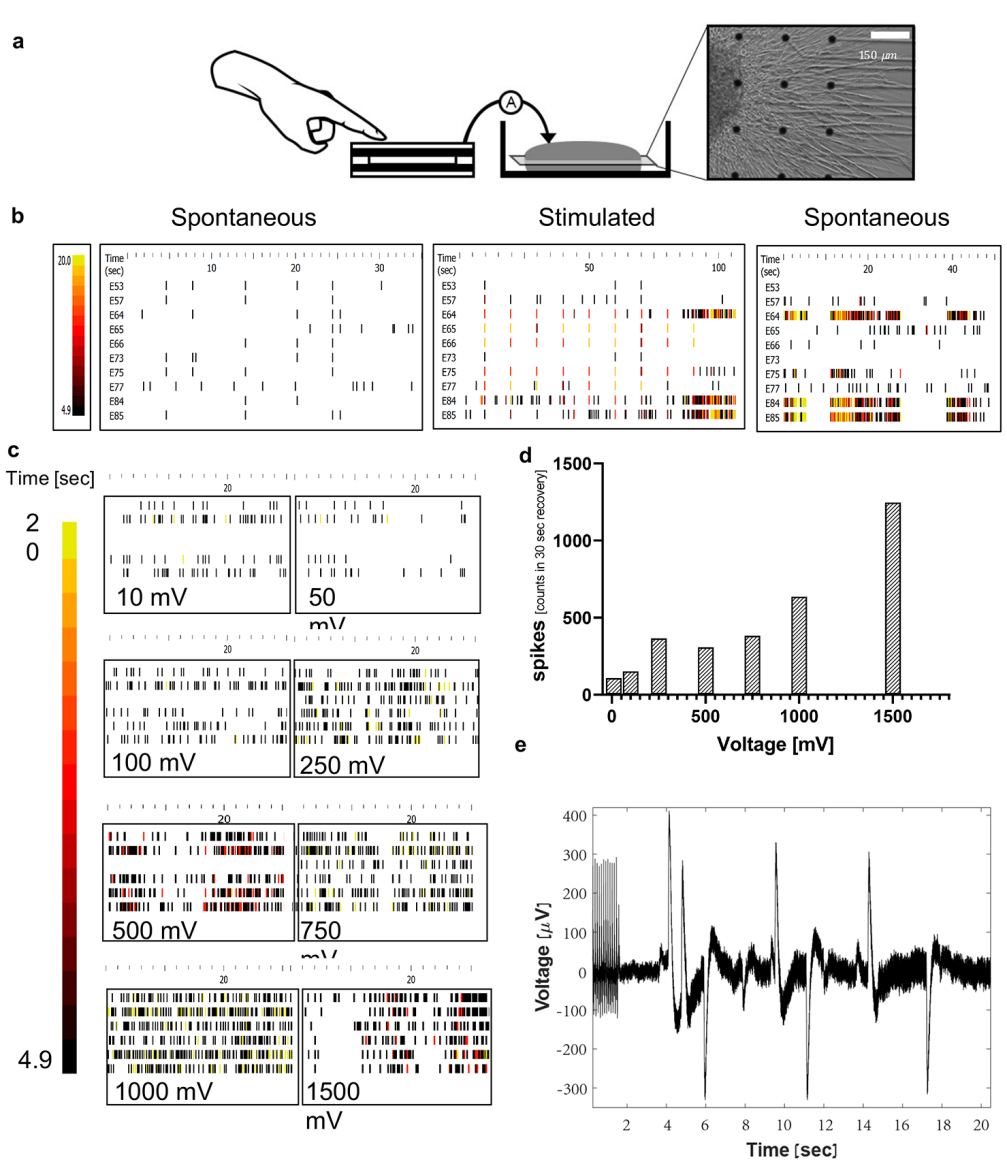
a. Schematic diagram of the *in vitro* setup: TENG-IT (5 mm × 5 mm) was connected to the MEA system, which stimulated the DRGs (scale: 0.3 mm). b. Raster plots of the electrophysiological response of the DRGs. The more lines and yellow appear, the higher the electrical activity. The plots of the 10 most active electrodes, before, during, and after stimulation of all electrodes with a priming set (10 repetitions of 1000 mV, 100 Hz pulses). c. Raster plot of the DRGs once they were stimulated with voltage proportional to the TENG-IT output for a specific pressure as measured in Figure 2d. d. Mean histogram of spikes per second, summation of all 6 electrodes in MEA plates (*n* = 4). e. Electrical response of the DRGs after they were directly stimulated by the TENG-IT platform.

Our next step, before *in vivo* validation, was to perform *in vitro* proof-of-principle, by characterizing the TENG-IT’s capacity to activate sensory neurons and, specifically, mouse dorsal root ganglia (DRGs). For these experiments, we developed a multielectrode array (MEA) platform that could be integrated with the TENG-IT device (TENG-IT surface area: 25 mm^2^ [5 mm × 5 mm]; Figure 4a, Figure S5). The MEA was used both to measure cells’ electrical activity and to generate potential that stimulates the cells.

DRGs are known to exhibit limited spontaneous electrophysiological activity *in vitro.*(37) Indeed, MEA measurements of the DRGs’ baseline activity did not show significant spontaneous electrical activity (Figure 4b, Figure S6). To verify that the cells were capable of electrical activity, we exposed them to KCl (in line with prior studies^37^) and observed that, as expected, electrical activity increased following KCl addition (Figure S6).

Preliminary experiments indicated that the DRG response to stimulation (electrical potential) generated by the TENG-IT improves when the cells are first primed to be electrically active, rather than stimulated from their baseline (inactive) state (data not shown). Because KCl exposure kills the cells, we evaluated the capacity of the MEA’s stimulation system to prime the DRGs in this manner (see Materials and Methods). We observed that, indeed, exposure to MEA stimulation elicited a significant increase in the DRGs’ spontaneous electrical activity (Figure 4b).

Next, we evaluated the DRGs’ capacity to respond differentially to different levels of electrical potential. This step was aimed at supporting a basic assumption underlying the TENG-IT concept, which is that neurons can sense different levels of electrical potential, generated by different levels of tactile pressure on the implanted TENG-IT (and ultimately transmit this information as tactile sensory information to the brain). In this experiment, we exposed the (electrically primed) DRGs to external electric potential, generated by the MEA itself, and proportional to the potential that was expected to be generated by the TENG-IT (as measured in Figure 2c,d). Quantification of the neuronal electrical activity (Figure 4c,d) showed that, indeed, the DRGs’ electrical activity was more intensive in the presence of higher levels of voltage used for DRG stimulation.

Our final step in the *in vitro* testing process was to expose (electrically primed) DRGs to direct stimulation from the TENG-IT (see Figures 4a and S5 for images of the experimental setup). As shown in Figure 4e, TENG-IT stimulation elicited electrical activity in the DRGs.

### TENG-IT Implantation Provides Tactile Sensation Capabilities and Does Not Interfere with Motor Abilities in Rats

#### Mapping of the Rat’s Sensory System

**Figure 5:**
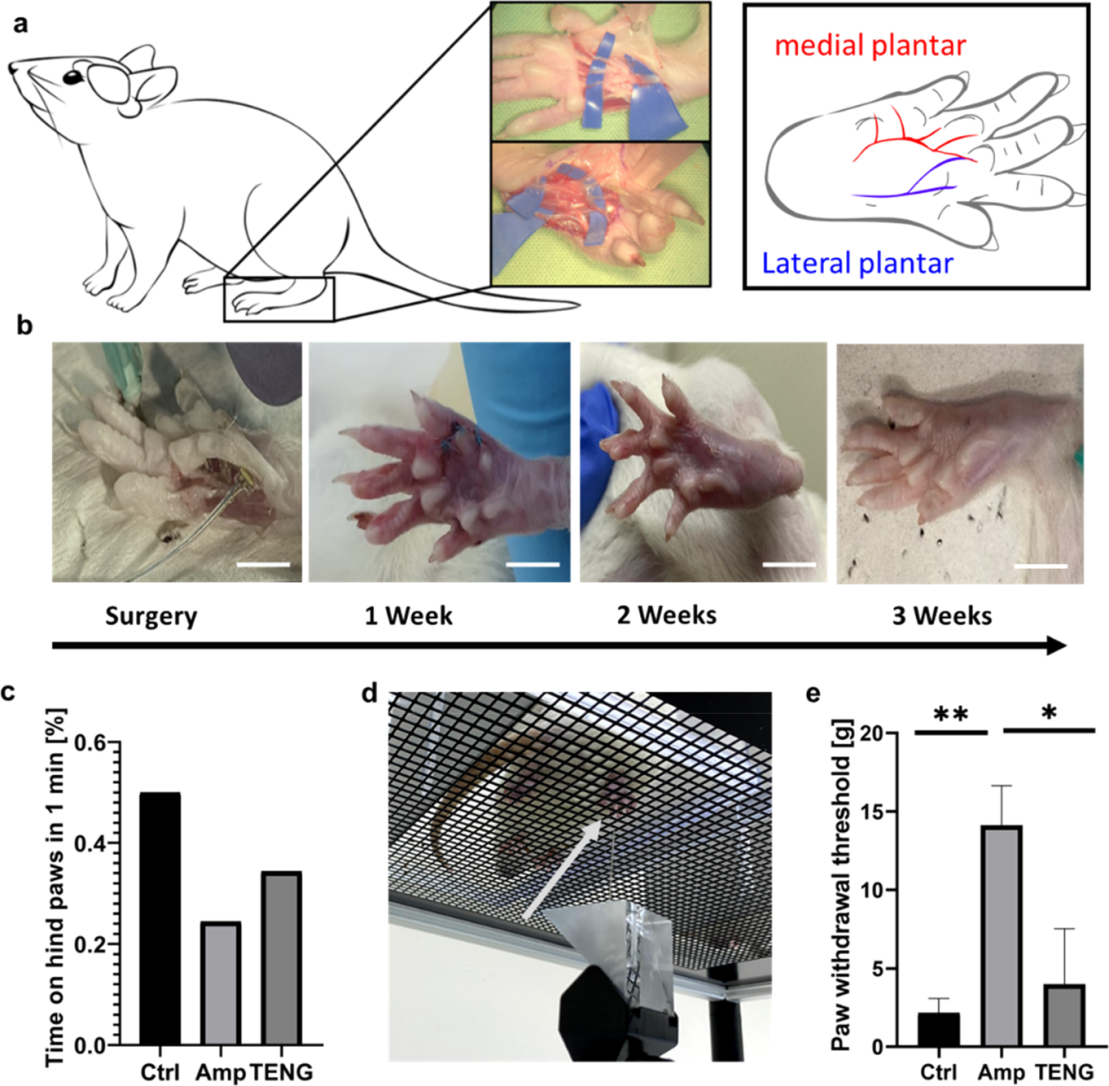
a. Schematic illustration based on anatomical dissection of the nerves of a Wistar rat’s hindfoot. b. Images of surgical implantation of TENG-IT (8 mm × 3 mm) in a rat’s left hindfoot, and post-operative recovery (scale = 1 cm). c. Percentage of time spent on hind paws for each group, over the course of 1 min. d. von Frey test setup. Arrow points to the tip of the device. e. Difference in average required peak force between the right and left hind paws in the von Frey test, measured for three experimental groups: control, amputee, TENG-IT (*P*-values: **p = 0.0099, *p = 0.0242).

To test and validate the TENG-IT, we used a rat model, as the anatomy of the rat tactile sensory system has been suggested to be very similar to that of the human sensory system.^38−41^ To obtain information necessary for device implantation, we performed a preliminary dissection to map the sensory nerve system of the rat hind paw (Figure 5a). Prior studies have indicated that sensation in the central plantar part of the rat hindfoot is transmitted by the tibial nerve, via the lateral and medial plantar branches, with additional minor contributions from the saphenous and sural nerves.^38−41^ In line with these findings, we observed in all procedures that hindfoot sensation was supplied mainly by the medial plantar branch of the distal tibial nerve, through multiple cutaneous branches (Figure 5a). This finding supports the likelihood that removal of a segment of the medial and lateral plantar nerves should anesthetize the hindfoot. Notably, the terminal part of the distal tibial nerve is not important for extrinsic motor function, suggesting that transection of the nerve should not interfere with the rat’s movement capabilities (Figure 5b,c, SI Movie 2).

#### Sensation Assessment: von Frey Test

We divided 12-week-old female Wistar rats (*n* = 9) into 3 groups: “control” (*n* = 3), in which no procedure was done; “amputee” (*n* = 3), in which a segment of the left distal tibial nerve was removed; and “TENG-IT” (*n* = 3), in which a segment of the left distal tibial nerve was removed, and a TENG-IT device (surface area: 8 mm × 3 mm) was implanted at the left hindfoot and connected with cuff electrodes to the terminal part of the remaining portion of the left distal tibial nerve (Figure 5b). The implanted device was encapsulated and sealed with biocompatible materials, to prevent inflammation. After the rats recuperated from surgery, we monitored their movement (measured according to the amount of time rats spent on their hindfeet over the course of 1 min) and observed no significant differences between the groups, suggesting that all animals were well and healthy, and their motor capability was not affected (Figure 5c, Figure S7, and SI Movie 2).

We used a von Frey test to measure the rats’ sensation^42^ (Figure 5d). In this setup, increasing force is applied to the rat’s paw from below, and once the rat senses the force, it lifts its paw. Rats with functional tactile sensation respond to low amounts of force, whereas rats lacking tactile sensation respond only to much higher levels of force. It should be noted that even rats in which the distal tibial nerve has been severed will eventually respond, as high levels of force can move the entire leg, and then the rat will notice the force that has been applied.

Since different animals are likely to have different thresholds for sensation, we tested both hindfeet (treated and untreated) as a within-subjects control, in addition to using a control group. Comparing measurements in the left hindfoot (*i.e*., the treated hindfoot in the amputee and TENG-IT groups), we observed that the control group responded to a low level of force (2.69 ± 0.12g), significantly lower than that required to elicit a response from the amputee group, which responded only to high levels of force (14.12 ± 2.53g) (Figure 5e). Rats in the TENG-IT group, in turn, responded to a much lower amount of force compared with the amputee group (3.99 ± 3.54g, *p*-value = 0.0099); this level was similar to the amount of force required for the control group (Figure 5e).

#### Immunohistochemistry

**Figure 6:**
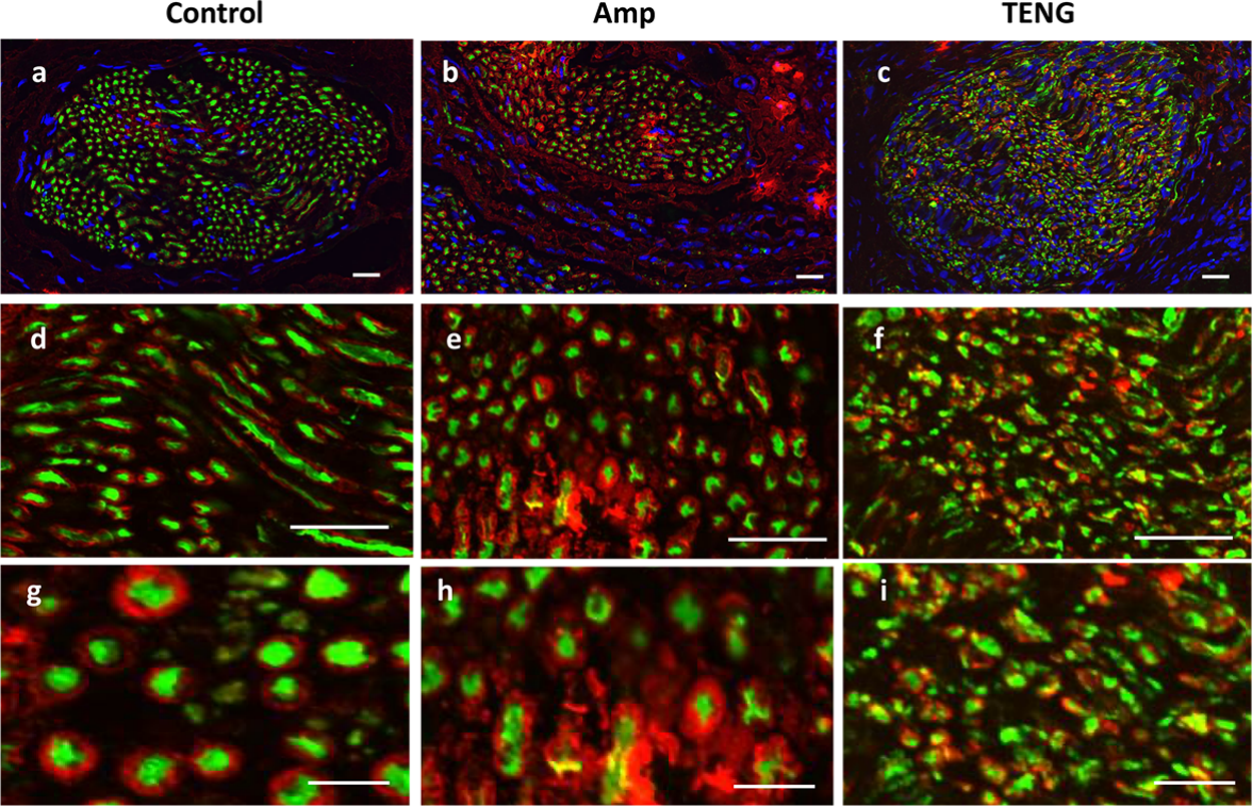
Immunohistochemistry of the sensory nerve for rats in the control, amputee (“Amp”), and TENG-IT (“TENG”) groups. To better characterize the nerve response to the transection and device implantation process, the nerve was stained for neurofilaments (NF, green), Myelin (MBP, red), and nucelli (DAPI, blue); scale bar: 20 μm for images a–f, and 10 μm for g–i.

In an effort to characterize the nerves’ response to the surgical procedure and to the activity of the TENG-IT, we sacrificed the rats and subsequently performed immunohistochemistry (IHC) experiments on rats in each group, measuring expression of neurofilaments (NF) and myelination (Myelin) in the distal tibial nerve proximal to the level of transection (Figure 6, Figure S8). As shown in Figure 6 and Figure S8, the amputee sample showed clusters of myelin that were absent from the control sample; similar clusters have previously been observed in studies of rats with damaged peripheral nervous systems (PNS).^43,44^ The characteristics of the TENG-IT sample were similar to those of the amputee sample, suggesting that both groups experienced similar nerve damage (due to the removal of part of the nerve). The fact that TENG-IT rats experienced nerve damage yet showed sensory capabilities similar to those of the control rats lends support to our assumptions regarding the TENG-IT’s mode of operation: namely, the cuff electrode attached to the TENG-IT bypasses the damaged area of the nerve and relays signals to the (healthy) nerve to which it is attached, enabling tactile sensation capabilities to be (at least to some extent) restored.

This work demonstrated a concept for an implanted device—the TENG-IT—with the potential to restore tactile sensation following peripheral nerve damage. The TENG-IT is a triboelectric device that is implanted under the skin and that translates tactile pressure into electrical potential, which it relays (via cuff electrodes) to healthy sensory nerves, thereby stimulating them to mimic tactile sensation. The device is self-powered and is made of biocompatible materials (CAB:PDMS). First, we demonstrated the device *in vitro,* showing that it has the capacity to elicit electrical activity in sensory neurons (DRGs) and that the extent of this activity is dependent on the level of pressure (electrical potential) applied, suggesting that the device can simulate actual tactile sensation. We subsequently demonstrated the functionality of the TENG-IT *in vivo*. Specifically, we implanted the device in rats in which a tactile sensory nerve (the left distal tibial nerve) had been severed, and showed that the device provided these rats with tactile sensation capabilities, as measured by a von Frey test. IHC examination enabled us to further characterize the biological response to nerve transection and device implantation and activation. Our observations support a process in which the TENG-IT enables tactile signals to bypass damaged areas (as reflected in myelin clusters) and to stimulate healthy nerves to which the device is attached.

The process of developing the TENG-IT produced several insights that may inform further development. For example, in evaluating biocompatible dielectric materials to use in the device, we found that CAB was preferable to nylon (for use as the positively charged material), as CAB was much more stable and produced higher output voltage. Moreover, in experiments in which we simulated large numbers of “finger taps” over time, we found that the electrical potential produced by the device increased over time after initial use, until it reached saturation. A similar pattern was observed when the device was placed in biological conditions over 26 days and subjected periodically to repeated application of pressure. We attributed the increase in electrical potential to an increase in the roughness of the material, which increased the active surface area and thus the output voltage.^45^ This characteristic should be taken into account in designing TENG-IT devices for medical use; for example, it may be beneficial, prior to implantation, to first activate the TENG-IT repeatedly until the output voltage reaches saturation. Alternatively, it is possible that the body might adapt on its own to the gradual increases in output voltage.

Another contribution of the current study is a more in-depth characterization of the rat’s tactile sensory system, toward the development of an applicable rat model for restoration of tactile sensation. Indeed, few animal models exist for this explicit purpose. Our work suggests that transection of the distal tibial nerve can serve as a useful model in these contexts.

To further adapt the TENG-IT device for medical use, several additional developments will be necessary. The first is miniaturization. The surface area of the current device is about 24 mm^2^, which may be somewhat cumbersome if the device is implanted, *e.g*., in a human fingertip. More broadly, it may be useful to create an array of TENG-IT devices of different sizes and with different levels of sensitivity for implantation in different parts of the body. Second, it will be necessary to refine the implantation procedure, taking into account the part of the body in which the device is to be implanted, the configurations of damaged and healthy nerves in the area, and the quantity of sensory neurons connected to the nerve. The third development is output adjustment, primarily for the purpose of matching the “natural feeling of sensation”. Notably, such adjustment may provide additional benefits, as recent literature suggests that appropriate stimulation of injured peripheral nerves can assist in nerve regeneration.^46^ Fourth, though we tested the durability of our device over half a million taps and in simulated biological conditions over 26 days, it is necessary to verify the long-term stability and functionality of the TENG-IT device *in vivo*, and to adjust its design as necessary. It should ideally be possible for the device to function for many years after implantation without the need for replacement.

## Conclusions

The potential of TENGs as a tool for harvesting biomechanical energy has been widely discussed,^22^ and prior studies have touched upon the TENG’s potential as a tool for aiding tactile sensation.^47,48^ In this work, we demonstrated an *in vitro* and *in vivo* proof-of-concept for the capacity of TENG technology to function as a simple, scalable, inexpensive, and self-powered device for tactile sensory restoration, with relatively few components and a straightforward fabrication process. If developed to its full potential, the TENG-IT may ultimately provide a means of restoring tactile sensation without the need for an external power source, in addition to overcoming some of the other drawbacks of existing neuro-prosthetic solutions (see Table S1 for a detailed comparison of the TENG-IT to alternative devices).

## Methods and Materials

### TENG-IT Manufacturing

In general, the TENG-IT, similarly to TENGs described in previous studies outside the biomedical domain,^25−27^ is composed of several different layers with different functions. The innermost layer is the friction layer that creates the triboelectric effect, which allows the device to be self-powered. These layers are separated by thin, flexible strips; these spacers allow contact and separation of the friction layers, thus creating the triboelectric charge. Each of the friction layers is attached to a conductive film, which functions as an electrode that facilitates charge transfer. The device is encapsulated in a biocompatible isolating material (PDMS and fibrin glue) that prevents contact with the surrounding physiological environment. We fabricated and tested devices of various sizes and shapes, ranging between 24 mm^2^ and 25 cm^2^. Ultimately, in the experiments for development and characterization of the TENG-IT, we used a device with surface area of 5 mm × 5 mm; in our *in vitro* experiments, we used a device with surface area of 5 mm × 5 mm; and in order to adjust the device for the *in vivo* experiments, the device was elongated to better fit the paw’s shape resulting in a rectangular surface area of 8 mm × 3 mm (24 mm^2^).

#### Gold Evaporation

Kapton strips (Ka) (Dupont, Delaware, USA) at a thickness of 125 μm served as the basis of the device structure, thus providing high flexibility and strength. A 5 nm adhesion layer of titanium (Ti) was evaporated by electron beam evaporation (VST, TFDS-870) on the Kapton strip. A 100 nm layer of gold (Au) was evaporated in the same way on top of the titanium layer serving as an electrode.

In our experiments for characterizing the effects of Kapton thickness on the TENG-IT’s performance, the “thick” layer of Kapton was 125 μm thick (catalog number: 677-930-79) and the “thin” layer of Kapton was 13 μm thick (catalog number: 488-784-98).

#### Adhesive Treatments

To improve attachment of the dielectric materials to the Au layer, we soaked Ka–Au strips for 30 min in mercaptohexadecanoic acid (MHDA; Sigma-Aldrich, Rehovot, Israel), diluted at a ratio of 1:100 in ethanol (Bio lab, Jerusalem, Israel), creating a self-assembled monolayer of thiols for better adhesion with Nylon-6-6 (Sigma-Aldrich, Rehovot, Israel) and CAB (Sigma-Aldrich, Rehovot, Israel).

#### Preparation of Dielectric Materials

PDMS (Sigma-Aldrich, Rehovot, Israel) was mixed at a ratio of 1:10 with curing agent (Sylgard 184, Sigma-Aldrich, Rehovot, Israel). Nylon-6-6 beads were dissolved in hexafluoro-2-propanol (HFIP; Sigma-Aldrich, Rehovot, Israel) at 60 mg/mL and sonicated at 45 °C for 30 min. CAB was dissolved in methyl isobutyl ketone (MIBK; Sigma-Aldrich, Rehovot, Israel) at 84 mg/mL. The solution was mixed until fully dissolved. CAB and Ny electrodes were soaked in MHDA solution (7.2 mg/mL in ethanol) to improve adhesion.

#### Spin Coating

The Ka–Au base was held by vacuum, and dielectric materials (PDMS, Ny, CAB) were spin-coated onto it (Ni-Lo Scientific, Ottawa, Canada) at rotation velocities of 1000, 1800, and 1800 rpm for PDMS, CAB, and Ny, respectively. Each spin coat lasted 60 s, and final rotation speed was achieved in acceleration of 200 rounds/s^2^. Large samples (when the TENG-IT is larger than 10 mm × 10 mm) were poured with 500 μL of the dielectric material in its liquid form and were used for initial characterization of the TENG-IT. Small samples (5 mm × 5 mm) were poured with 200 μL of the liquid dielectric coating.

#### Curing

PDMS-coated Ka–Au strips were cured overnight (12 h) at 60 °C (ThermoFisher Scientific, Kiriyat Shemona, Israel). Ny and CAB Ka–Au strips were allowed to air-dry and attach overnight before device assembly and subsequent measurements.

#### Wiring

The exposed Au surface area was kept clear from the coating using elastic one-sided duct tape. Once the dielectric material was fully attached and cured, the duct tape was removed, and a copper wire with an exposed edge was connected using either fast-drying silver or gold paint (Ted Pella, Redding, California, United States) applied to the open Au area.

#### Spacing

Thin strips of Very High Bond tape (VHB; 3M, Teva Pharmaceuticals, Shoham, Israel) at varying thicknesses (0, 125, 250, 500, 750, 1000, 1500, and 2000 μm) were placed on top of the PDMS layer at each side of the upper surface area of the TENG-IT. The other side of the TENG-IT, containing the Ny/CAB layer, was inverted and placed directly above the PDMS layer (and separated at the sides by the VHB strips). The final height was determined according to Figure S3, where 750 μm demonstrated the optimal configuration.

#### Encapsulation

The TENG-IT was enclosed from both sides by a thin layer of 125 μm 3M VHB, and the edges were reinforced using Fibrin glue (Evicel - Omrix Biopharmaceuticals, J&J, Ness Ziona, Israel).

### Device Characterization

#### Linear Motor for TENG Pressure

Controlled finger tapping was mimicked by an industrial linear motor equipped with a Hall sensor feedback system (Faulhaber, Hx 3600, Schönaich, Germany). Electricity for the system was supplied by a connection to a 12 V standard power supplier. The motor was fixed to a custom-designed stage built of aluminum, allowing accurate positioning. Motor control (and applied pressures on the device, accordingly) was configured using EasyMotion Studio software (Faulhaber, Schönaich, Germany). A trapezoidal repeated loop was coded, producing ongoing and continuous tapping of the motor. Basic movement was set from a height of 0 mm (full contact with TENG-IT) up to 14 mm (no contact at all). The pressure was calculated using eq 2.
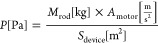
2where *M*_rod_ stands for the mass of the linear motor rod and head caps, as measured experimentally (105 g); *A*_motor_ is the acceleration downward of the rod as set in the software; and *S*_device_ is the device surface area upon which the pressure was applied.

#### Recordings

The two TENG-IT electrodes were connected to a V/I reader (NI6003; National Instruments [NI], Holon, Israel). Open circuit voltage was measured and recorded using the NI DAQExpress 2.0 software. Sampling rate was set to 25,000 Hz. Records of the TENG output voltage were analyzed using a custom-designed MATLAB code.

#### TENG-IT Data Analysis

All experiments were carried out three times. Records of 30 s of the TENG-IT output voltage were exported in .csv format and analyzed using a custom-designed MATLAB code.

#### TENG-IT Characterization in Dry Conditions

Encapsulated TENG-IT devices were placed in a Petri dish over 36 h. The motor tapped at a frequency of 4.5 Hz continuously.

#### TENG-IT Characterization in Biological Conditions

Encapsulated TENG-IT devices were placed in a Petri dish, covered with PBS, and placed in a biological incubator at 37 °C for 26 days. In each measurement, the motor tapped at a frequency of 1 Hz for 30 min. Statistical analysis was done on 30 s recordings after the 30 min tapping period.

### In Vitro DRG Activation

#### MEA Coating Plates

MEA plates (Multi Channel Systems 200/30; Multi Channel Systems, Reutlingen, Germany) were ozonized for 30 min under UV light sterilization. A day before cell culturing, plates were coated with 500 μL poly(d-lysine) (PDL) (Sigma-Aldrich, Rehovot, Israel), mixed with PBS (Sigma-Aldrich, Rehovot, Israel) at 1:50, and incubated overnight. On the day of seeding, PDL was removed, and plates were washed three times with PBS before being coated with 1 mL laminin solution (20 μg/mL in plating medium) (Sigma-Aldrich, Rehovot, Israel). Plates were incubated for 2 h before seeding, and were washed twice with PBS before seeding.

#### DRG Extraction and Cell Seeding

ICR mouse embryos (Envigo Laboratories, Jerusalem, Israel) at E12.5 were iced and then soaked in Hanks’ Balanced Salt solution (HBSS; Sigma-Aldrich, Rehovot, Israel). The spinal meninges was separated according to the protocol described thoroughly by Perlson et al.^49^ DRG explants were isolated from the meninges by surgery under the microscope from L1 to L9 and placed at the center of the MEA plate (1 explant for each plate, approximately 2000–3000 cells). Explants were covered with 5 μL of Matrigel (Corning, New York, United States) enriched with aqueous nerve growth factor (NGF) solution (Alomone laboratories, Jerusalem Israel) at 5 μg/mL. The ratio between the NGF solution and the Matrigel was 1:100, in order to make sure the explants were tight and as close to the electrodes as possible. Explants were placed in an incubator (37 °C, 5% CO_2_) for 30 min before adding 2 mL of culture medium to each plate. The medium was composed of 96% Neurobasal (Gibco, ThermoFisher Scientific, Rhenium, Modi’in-Maccabim-Re’ut, Israel), 2% B-27 (Gibco, Rhenium, Modi’in-Maccabim-Re’ut, Israel), 1% penicillin–streptomycin (Sigma-Aldrich, Rehovot, Israel), and 1% GlutaMAX (Gibco, ThermoFisher Scientific, Rhenium, Modi’in-Maccabim-Re’ut, Israel). The medium was freshly enriched with 5 μL/mL NGF on the day of the medium addition/replacement. Medium was replaced on the day following the seeding and once every 2 days thereafter. Electrical activity measurements were done at DIV 4.

#### Electrical Activity Measurements

MEA plates were inserted into the mini-MEA reader (Multi Channel Systems) while inside the incubator. The reader was connected to the recording computer through a designated adapter and controller (Multi Channel Systems). Recordings were done at a sample rate of 10,000 Hz. For each plate or sample, baseline activity was recorded for 5 min. Then, each MEA plate was exposed to stimulation (either chemical stimulation, electrical stimulation via the MEA, or TENG-IT stimulation; see below), and the 6–10 most active electrodes in the plate were analyzed.

#### MEA Data Analysis

Raw data from all electrodes were recorded in Multi Channel Systems Experimenter software. Further analysis was done using NeuroExplorer software. Data were band-pass filtered in the neuronal range of activity (200–4000 Hz). Spike detection was applied for 4 standard deviations lower and upper edge thresholds. Raster plots and rate histograms were generated for the 6 most active electrodes that had visible axonal growth/cell body presence in their area.

#### Chemical Stimulation

For each plate, 50 mM potassium chloride (KCl; Sigma-Aldrich, Rehovot, Israel) was added to the culture medium. Recording was stopped after 2 min or when there was no observed electrical activity, the later of the two.

#### Electrical Stimulation/Priming via the MEA

Stimulation of the DRGs was done by the inner signal collector unit (SCU) stimulator function provided by the MEA system’s software (Experimenter Software, Multi Channel Systems). All 60 electrodes were used as active stimulators. A “priming set” of stimuli was applied to each culture. The priming set consisted of a sequence of 10 bursts of pulses, with intervals of 10 s between consecutive bursts. Each burst consisted of 10 repetitions of a 1000 mV pulse with a pulse width of 750 μs, and at a frequency of 100 Hz. The total duration of the priming set was 100 s. This priming procedure is based on earlier studies regarding stimulation of DRGs on MEA.^37^ Recording was stopped after 5 min or when there was no observed electrical activity (1 or less observed spike per minute), the later of the two.

In our experiments aimed at assessing DRG response to varying levels of voltage, stimulations at increasing amplitude of voltage were applied (10, 50, 100, 250, 500, 750, 1000, and 1500 mV accordingly). Each sample was primed once for 10 repetitions of 1000 mV, 100 HZ pulses. Next, the stimulation began with a 2 min baseline recording.

#### TENG-IT Stimulation

After undergoing the MEA priming procedure outlined above, the DRGs were stimulated by the TENG-IT, which was connected to the MEA via metal cables. The TENG-IT was set to 10 stimulations per second for 0.5 s.

### TENG Implantation in Rats and Evaluation of Tactile Sensation

#### Device Preparation

The TENG-IT devices designated for implantation each had a surface area of 8 mm × 3 mm; the average peak-to-peak voltage output that was measured prior to implantation was 1.0–1.5 V. All devices were sterilized with ethanol and PBS prior to the implantation process.

#### Animals and Sequence of Procedures

Female Wistar rats (12 weeks old; *n* = 9) were obtained from Envigo Israel and housed with a 12 h light/dark cycle and were fed with autoclaved rodent pellet (Koffolk 19–510; Koffolk Ltd., Petach Tikva, Israel) and sterile water ad libitum. All experimental procedures were approved by the Tel Aviv University Animal Care Committee according to national guidelines (permit #01-19-029).

Prior to initiation of the surgical procedures, all rats (control, amputee, and TENG-IT groups) were placed in the animal house for a week for an adjustment period. Rats that underwent implantation surgery recovered for 10 days prior to the von Frey test. The von Frey test was performed on each rat approximately every 3–4 days, over the course of 19 days (5 measurements per rat). Afterward, the animals were sacrificed, and their tissues were fixed and stained for immunohistochemistry.

#### Surgical Nerve Mapping

An inverted L incision was made in the hind paw, on the lateral side. The skin flap was elevated from lateral to medial. Sensory branches to the skin were cut. The distal tibial nerve and its branches were identified and dissected. In all hind paws, medial and lateral plantar nerves were observed. We observed that the medial plantar nerve had sensory branches in most of the central part of the paw, with contribution of the lateral plantar nerve on the lateral side. The medial tibial branch was branched across the medial 4 digits, and the lateral plantar nerve branch was branched across the lateral 2 digits.

#### Surgical Procedures: Sensory Nerve Transection and TENG-IT Implantation

For surgery, rats in the amputee and TENG-IT groups were anesthetized, and then an incision was made in the lateral part of the left posterior foot (as described earlier), and a segment of the medial and lateral tibial nerves was removed.

For rats in the TENG-IT group, the TENG-IT was placed subcutaneously in the central part of the rat’s paw and then attached to the terminal part of the transected tibial nerve using a cuff electrode (2 Micro Cuff Tunnel 0,0000 1.556,70 200/Pt–Ir/2 mm long/0.5 mm C2C/0.2 × 0.5 mm O/Cable 30 cm entry lateral – weld tube 001; CorTec, Saint Paul, Minnesota, United States). A 9-0 nylon suture was used to secure the positioning and the attachment of the cuff to the nerve.

The surgical incision was sutured with a 5-0 nylon suture, and the rats were treated with antibiotics and painkillers (Rymadil; Vetmarket, Shoham, Israel). After the surgery, the rats recovered for 10 days before the von Frey tests.

#### Post-Op Care

The rats were placed in Tel Aviv University’s animal care facility and allowed to move as tolerated with no immobilization. Each rat wore a protective collar on its neck for 3 days post-op to prevent the rat from damaging the healing wound. Pain medication and antibiotics were administered during the first week of recovery. Wound recovery and weight were assessed daily. After approximately 10 days, full recovery of the surgical wound was observed.

#### Post-Recovery Movement Assessment

To assess rats’ movement following the surgical procedure, we filmed all rats, including those in the control group, and quantified the amount of time each rat spent on its hind paws over the course of a 1 min video.

#### Von Frey Test

The von Frey apparatus (Ugo Basile, Italy) consists of an elevated horizontal wire mesh stand. The rat stands on top of the wire mesh, inside a plexiglass enclosure with an open bottom. Pressure is applied to the rat’s paw from below, using a tip. When the rat lifts its foot, indicating that it has sensed the pressure applied,^50^ the maximal force applied is automatically recorded by an electronic device. Each rat underwent the von Frey test once every 3 or 4 days, over the course of 19 days, as noted above. Each animal was subjected to 5 measurements in each hind leg, on each measurement day.

#### Statistics

All experiments were carried out at *n* = 3–4 per condition (repetitions and samples/cohorts). Prism software (GraphPad Software, La Jolla California USA) was used for one-way ANOVA with a Sidak post-test and for two-way ANOVA, analyzed with a Tukey post-test. A nonpaired Student’s *t* test was used to test the significance of differences between conditions.

#### Immunohistochemistry

Nerve tissues were fixed in 4% paraformaldehyde (PFA) solution (Bio lab, Jerusalem, Israel), dehydrated in increasing concentrations of ethanol and xylene, embedded in paraffin, and sectioned to 5 μm. Deparaffinized and rehydrated sections were incubated for antigen retrieval with 0.01 M sodium citrate in ddH_2_O, pH 6.0, followed by extensive rinses in ddH_2_O, and blocked with 10% normal goat serum (Jackson Laboratory, Bar Harbor, Maine, United States) in PBS containing 0.1% bovine serum albumin (BSA; Sigma-Aldrich, Rehovot, Israel). Sections were incubated overnight at room temperature in a humidified chamber with either rabbit polyclonal Anti-Neurofilament heavy polypeptide antibody (Abcam, Zotal, Tel Aviv, Israel) (diluted 1:200) or mouse monoclonal Anti-Myelin Basic Protein antibody (Abcam, Zotal, Tel Aviv, Israel) (diluted 1:200). Secondary antibodies Alexa goat anti-mouse 594 and goat anti-rabbit 488 (Invitrogen, Rhenium, Modi’in-Maccabim-Re’ut, Israel), diluted 1:750, were used as secondary antibodies and incubated for 1 h. Sections were washed with PBS and mounted with DAPI Fluoromount G (SouthernBiotech, Birmingham, Alabama, United States). Negative controls were incubated only with secondary antibodies and with only one primary antibody, followed by both secondary antibodies for IF double-staining experiments. Image visualization was performed on an Olympus FV3000 confocal microscope, with a UCPLFLN × 20 objective/0.7 NA. The acquisition and analysis software program was FLUOVIEW.

## Supporting Information Available

Figure S1: SEM imaging of TENG-IT dielectric materials. Figure S2: Nylon as a dielectric material. Figure S3: effect of the “spacer” thickness on the TENG-IT output voltage. Figure S4: effect of the Kapton thickness on the TENG-IT output voltage. Figure S5: detailed description of the in vitro TENG-IT assessment platform. Figure S6: electrical activity validation test of the TENG-IT using the in vitro assessment platform. Figure S7: Von Frey test setup. Figure S8: immunohistochemistry of the sensory nerve. Table S1: Technologies comparison table (PDF)
Movie S1: Triboelectric response of the TENG-IT (MP4)
Movie S2: Typical rat movement after the removal of the sensory nerve (MP4)

## References

[ref1] Noble J.; Munro C. A.; Prasad V. S.; Midha R. Analysis of Upper and Lower Extremity Peripheral Nerve Injuries in a Population of Patients with Multiple Injuries. J. Trauma 1998, 45 (1), 116–122. 10.1097/00005373-199807000-00025. 9680023

[ref2] Novak C. B.; Anastakis D. J.; Beaton D. E.; Mackinnon S. E.; Katz J. Relationships among Pain Disability, Pain Intensity, Illness Intrusiveness, and Upper Extremity Disability in Patients with Traumatic Peripheral Nerve Injury. J. Hand Surg Am. 2010, 35 (10), 1633–1639. 10.1016/j.jhsa.2010.07.018. 20888499

[ref3] Ciaramitaro P.; Mondelli M.; Logullo F.; Grimaldi S.; Battiston B.; Sard A.; Scarinzi C.; Migliaretti G.; Faccani G.; Cocito D.; Traumatic Peripheral Nerve Injuries: Epidemiological Findings, Neuropathic Pain and Quality of Life in 158 Patients. J. Peripher. Nerv. Syst. 2010, 15 (2), 120–127. 10.1111/j.1529-8027.2010.00260.x. 20626775

[ref4] Ring D. Symptoms and Disability after Major Peripheral Nerve Injury. Hand Clin 2013, 29 (3), 421–425. 10.1016/j.hcl.2013.04.008. 23895722

[ref5] Phillips C.; Blakey G.; Essick G. K. Sensory Retraining: A Cognitive Behavioral Therapy for Altered Sensation. Atlas Oral Maxillofac Surg Clin North Am. 2011, 19 (1), 109–118. 10.1016/j.cxom.2010.11.006. 21277504 PMC3073500

[ref6] Dalamagkas K.; Tsintou M.; Seifalian A. Advances in Peripheral Nervous System Regenerative Therapeutic Strategies: A Biomaterials Approach. Mater. Sci. Eng., C 2016, 65, 425–432. 10.1016/j.msec.2016.04.048. 27157770

[ref7] Elkwood A. I.; Holland N. R.; Arbes S. M.; Rose M. I.; Kaufman M. R.; Ashinoff R. L.; Parikh M. A.; Patel T. R. Nerve Allograft Transplantation for Functional Restoration of the Upper Extremity: Case Series. J. Spinal Cord Med. 2011, 34 (2), 241–247. 10.1179/107902611X12972448729521. 21675363 PMC3066509

[ref8] Mackinnon S. E.; Doolabh V. B.; Novak C. B.; Trulock E. P. Clinical Outcome Following Nerve Allograft Transplantation. Plast Reconstr Surg 2001, 107 (6), 1419–1429. 10.1097/00006534-200105000-00016. 11335811

[ref9] Ruijs A. C. J.; Jaquet J.-B.; Kalmijn S.; Giele H.; Hovius S. E. R. Median and Ulnar Nerve Injuries: A Meta-Analysis of Predictors of Motor and Sensory Recovery after Modern Microsurgical Nerve Repair. Plast Reconstr Surg 2005, 116 (2), 484–494. 10.1097/01.prs.0000172896.86594.07. 16079678

[ref10] Chortos A.; Liu J.; Bao Z. Pursuing Prosthetic Electronic Skin. Nat. Mater. 2016, 15 (9), 937–950. 10.1038/nmat4671. 27376685

[ref11] Yang J. C.; Mun J.; Kwon S. Y.; Park S.; Bao Z.; Park S. Electronic Skin: Recent Progress and Future Prospects for Skin-Attachable Devices for Health Monitoring, Robotics, and Prosthetics. Adv. Mater. 2019, 31 (48), 1904765 10.1002/adma.201904765. 31538370

[ref12] Cuberovic I.; Gill A.; Resnik L. J.; Tyler D. J.; Graczyk E. L. Learning of Artificial Sensation Through Long-Term Home Use of a Sensory-Enabled Prosthesis. Front. Neurosci. 2019, 13, 1 10.3389/fnins.2019.00853. 31496931 PMC6712074

[ref13] Ghomian T.; Mehraeen S. Survey of Energy Scavenging for Wearable and Implantable Devices. Energy 2019, 178, 33–49. 10.1016/j.energy.2019.04.088.

[ref14] Ward-Cherrier B.; Pestell N.; Lepora N. F. NeuroTac: A Neuromorphic Optical Tactile Sensor Applied to Texture Recognition. IEEE International Conference on Robotics and Automation (ICRA) 2020, 2654–2660. 10.1109/ICRA40945.2020.9197046.

[ref15] Ganzer P. D.; Colachis S. C.; Schwemmer M. A.; Friedenberg D. A.; Dunlap C. F.; Swiftney C. E.; Jacobowitz A. F.; Weber D. J.; Bockbrader M. A.; Sharma G. Restoring the Sense of Touch Using a Sensorimotor Demultiplexing Neural Interface. Cell 2020, 181 (4), 763–773. 10.1016/j.cell.2020.03.054. 32330415

[ref16] Pasluosta C.; Kiele P.; Stieglitz T. Paradigms for Restoration of Somatosensory Feedback *via* Stimulation of the Peripheral Nervous System. Clin. Neurophysiol. 2018, 129 (4), 851–862. 10.1016/j.clinph.2017.12.027. 29343415

[ref17] Resnik L.; Meucci M. R.; Lieberman-Klinger S.; Fantini C.; Kelty D. L.; Disla R.; Sasson N. Advanced Upper Limb Prosthetic Devices: Implications for Upper Limb Prosthetic Rehabilitation. Arch. Phys. Med. Rehabil. 2012, 93 (4), 710–717. 10.1016/j.apmr.2011.11.010. 22464092

[ref18] Nyska A.; Schiffenbauer Y. S.; Brami C. T.; Maronpot R. R.; Ramot Y. Histopathology of Biodegradable Polymers: Challenges in Interpretation and the Use of a Novel Compact MRI for Biocompatibility Evaluation. Polym. Adv. Technol. 2014, 25 (5), 461–467. 10.1002/pat.3238.

[ref19] Dellon E. S.; Mourey R.; Dellon A. L. Human Pressure Perception Values for Constant and Moving One- and Two-Point Discrimination. Plast. Reconstr. Surg. 1992, 90 (1), 112–117. 10.1097/00006534-199207000-00017. 1615069

[ref20] Zhu G.; Peng B.; Chen J.; Jing Q.; Lin Wang Z. Triboelectric Nanogenerators as a New Energy Technology: From Fundamentals, Devices, to Applications. Nano Energy 2015, 14, 126–138. 10.1016/j.nanoen.2014.11.050.

[ref21] Zhu G.; Lin Z.-H.; Jing Q.; Bai P.; Pan C.; Yang Y.; Zhou Y.; Wang Z. L. Toward Large-Scale Energy Harvesting by a Nanoparticle-Enhanced Triboelectric Nanogenerator. Nano Lett. 2013, 13 (2), 847–853. 10.1021/nl4001053. 23360548

[ref22] Zheng Q.; Zou Y.; Zhang Y.; Liu Z.; Shi B.; Wang X.; Jin Y.; Ouyang H.; Li Z.; Wang Z. L. Biodegradable Triboelectric Nanogenerator as a Life-Time Designed Implantable Power Source. Science Advances 2016, 2 (3), e1501478 10.1126/sciadv.1501478. 26973876 PMC4783121

[ref23] Wang Z. L. Triboelectric Nanogenerators as New Energy Technology for Self-Powered Systems and as Active Mechanical and Chemical Sensors. ACS Nano 2013, 7 (11), 9533–9557. 10.1021/nn404614z. 24079963

[ref24] Kaur N.; Pal K. Triboelectric Nanogenerators for Mechanical Energy Harvesting. Energy Technology 2018, 6 (6), 958–997. 10.1002/ente.201700639.

[ref25] Chen C.; Wei A.; Xie X.; Zhai N.; Wei X.; Peng M.; Liu Y.; Sun X.; Yeow J. Self-Powered on-Line Ion Concentration Monitor in Water Transportation Driven by Triboelectric Nanogenerator. Nano Energy 2019, 62, 442 10.1016/j.nanoen.2019.05.029.

[ref26] Zhai N.; Wen Z.; Chen X.; Wei A.; Sha M.; Fu J.; Liu Y.; Zhong J.; Sun X. Blue Energy Collection toward All-Hours Self-Powered Chemical Energy Conversion. Adv. Energy Mater. 2020, 10 (33), 2001041 10.1002/aenm.202001041.

[ref27] Chen C.; Guo H.; Chen L.; Wang Y.-C.; Pu X.; Yu W.; Wang F.; Du Z.; Wang Z. L. Direct Current Fabric Triboelectric Nanogenerator for Biomotion Energy Harvesting. ACS Nano 2020, 14 (4), 4585–4594. 10.1021/acsnano.0c00138. 32181639

[ref28] Zheng Q.; Shi B.; Fan F.; Wang X.; Yan L.; Yuan W.; Wang S.; Liu H.; Li Z.; Wang Z. L. *In Vivo* Powering of Pacemaker by Breathing-Driven Implanted Triboelectric Nanogenerator. Adv. Mater. 2014, 26 (33), 5851–5856. 10.1002/adma.201402064. 25043590

[ref29] Pu X.; Liu M.; Chen X.; Sun J.; Du C.; Zhang Y.; Zhai J.; Hu W.; Wang Z. L. Ultrastretchable, Transparent Triboelectric Nanogenerator as Electronic Skin for Biomechanical Energy Harvesting and Tactile Sensing. Science Advances 2017, 3 (5), e1700015 10.1126/sciadv.1700015. 28580425 PMC5451198

[ref30] Chen X.; Xie X.; Liu Y.; Zhao C.; Wen M.; Wen Z. Advances in Healthcare Electronics Enabled by Triboelectric Nanogenerators. Adv. Funct. Mater. 2020, 30 (43), 2004673 10.1002/adfm.202004673.

[ref31] Lin Z.; Yang J.; Li X.; Wu Y.; Wei W.; Liu J.; Chen J.; Yang J. Large-Scale and Washable Smart Textiles Based on Triboelectric Nanogenerator Arrays for Self-Powered Sleeping Monitoring. Adv. Funct. Mater. 2018, 28 (1), 1704112 10.1002/adfm.201704112.

[ref32] Guo H.; Pu X.; Chen J.; Meng Y.; Yeh M.-H.; Liu G.; Tang Q.; Chen B.; Liu D.; Qi S.; Wu C.; Hu C.; Wang J.; Wang Z. L. A Highly Sensitive, Self-Powered Triboelectric Auditory Sensor for Social Robotics and Hearing Aids. Science Robotics 2018, 3 (20), eaat2516 10.1126/scirobotics.aat2516. 33141730

[ref33] Fan F.-R.; Tian Z.-Q.; Lin Wang Z. Flexible Triboelectric Generator. Nano Energy 2012, 1 (2), 328–334. 10.1016/j.nanoen.2012.01.004.

[ref34] Wu C.; Wang A. C.; Ding W.; Guo H.; Wang Z. L. Triboelectric Nanogenerator: A Foundation of the Energy for the New Era. Adv. Energy Mater. 2019, 9 (1), 1802906 10.1002/aenm.201802906.

[ref35] Lin L.; Xie Y.; Wang S.; Wu W.; Niu S.; Wen X.; Wang Z. L. Triboelectric Active Sensor Array for Self-Powered Static and Dynamic Pressure Detection and Tactile Imaging. ACS Nano 2013, 7 (9), 8266–8274. 10.1021/nn4037514. 23957827

[ref36] Sriphan S.; Vittayakorn N. Facile Roughness Fabrications and Their Roughness Effects on Electrical Outputs of the Triboelectric Nanogenerator. Smart Mater. Struct. 2018, 27 (10), 105026 10.1088/1361-665X/aadb65.

[ref37] Newberry K.; Wang S.; Hoque N.; Kiss L.; Ahlijanian M. K.; Herrington J.; Graef J. D. Development of a Spontaneously Active Dorsal Root Ganglia Assay Using Multiwell Multielectrode Arrays. J. Neurophysiol. 2016, 115 (6), 3217–3228. 10.1152/jn.01122.2015. 27052585 PMC4946598

[ref38] Kambiz S.; Baas M.; Duraku L. S.; Kerver A. L.; Koning A. H. J.; Walbeehm E. T.; Ruigrok T. J. H. Innervation Mapping of the Hind Paw of the Rat Using Evans Blue Extravasation, Optical Surface Mapping and CASAM. J. Neurosci. Methods 2014, 229, 15–27. 10.1016/j.jneumeth.2014.03.015. 24721825

[ref39] Walcher J.; Ojeda-Alonso J.; Haseleu J.; Oosthuizen M. K.; Rowe A. H.; Bennett N. C.; Lewin G. R. Specialized Mechanoreceptor Systems in Rodent Glabrous Skin. J. Physiol. 2018, 596 (20), 4995–5016. 10.1113/JP276608. 30132906 PMC6187043

[ref40] Duraku L. S.; Hossaini M.; Hoendervangers S.; Falke L. L.; Kambiz S.; Mudera V. C.; Holstege J. C.; Walbeehm E. T.; Ruigrok T. J. H. Spatiotemporal Dynamics of Re-Innervation and Hyperinnervation Patterns by Uninjured CGRP Fibers in the Rat Foot Sole Epidermis after Nerve Injury. Mol. Pain 2012, 8, 61 10.1186/1744-8069-8-61. 22935198 PMC3492210

[ref41] Siegel S. M.; Lee J. W.; Oaklander A. L. Needlestick Distal Nerve Injury in Rats Models Symptoms of Complex Regional Pain Syndrome. Anesth. Analg. 2007, 105 (6), 1820–1829. 10.1213/01.ane.0000295234.21892.bc. 18042888

[ref42] Detloff M. R.; Clark L. M.; Hutchinson K. J.; Kloos A. D.; Fisher L. C.; Basso D. M. Validity of Acute and Chronic Tactile Sensory Testing after Spinal Cord Injury in Rats. Exp. Neurol. 2010, 225 (2), 366–376. 10.1016/j.expneurol.2010.07.009. 20643128 PMC4933012

[ref43] Guilbaud G.; Gautron M.; Jazat F.; Ratinahirana H.; Hassig R.; Hauw J. J. Time Course of Degeneration and Regeneration of Myelinated Nerve Fibres Following Chronic Loose Ligatures of the Rat Sciatic Nerve: Can Nerve Lesions Be Linked to the Abnormal Pain-Related Behaviours?. Pain 1993, 53 (2), 147–158. 10.1016/0304-3959(93)90074-Y. 8393169

[ref44] Nuytten D.; Kupers R.; Lammens M.; Dom R.; Van Hees J.; Gybels J. Further Evidence for Myelinated as Well as Unmyelinated Fibre Damage in a Rat Model of Neuropathic Pain. Exp. Brain Res. 1992, 91 (1), 73–78. 10.1007/BF00230014. 1338718

[ref45] Das P. S.; Chhetry A.; Maharjan P.; Rasel M. S.; Park J. Y. A Laser Ablated Graphene-Based Flexible Self-Powered Pressure Sensor for Human Gestures and Finger Pulse Monitoring. Nano Res. 2019, 12 (8), 1789 10.1007/s12274-019-2433-5.

[ref46] Zuo K. J.; Gordon T.; Chan K. M.; Borschel G. H. Electrical Stimulation to Enhance Peripheral Nerve Regeneration: Update in Molecular Investigations and Clinical Translation. Exp. Neurol. 2020, 332, 113397 10.1016/j.expneurol.2020.113397. 32628968

[ref47] Cai Y.-W.; Zhang X.-N.; Wang G.-G.; Li G.-Z.; Zhao D.-Q.; Sun N.; Li F.; Zhang H.-Y.; Han J.-C.; Yang Y. A Flexible Ultra-Sensitive Triboelectric Tactile Sensor of Wrinkled PDMS/MXene Composite Films for E-Skin. Nano Energy 2021, 81, 105663 10.1016/j.nanoen.2020.105663.

[ref48] Rao J.; Chen Z.; Zhao D.; Ma R.; Yi W.; Zhang C.; Liu D.; Chen X.; Yang Y.; Wang X.; Wang J.; Yin Y.; Wang X.; Yang G.; Yi F. Tactile Electronic Skin to Simultaneously Detect and Distinguish between Temperature and Pressure Based on a Triboelectric Nanogenerator. Nano Energy 2020, 75, 105073 10.1016/j.nanoen.2020.105073.

[ref49] Gluska S.; Chein M.; Rotem N.; Ionescu A.; Perlson E. Tracking Quantum-Dot Labeled Neurotropic Factors Transport along Primary Neuronal Axons in Compartmental Microfluidic Chambers. Methods Cell Biol. 2016, 131, 365–387. 10.1016/bs.mcb.2015.06.016. 26794524

[ref50] Ferrier J.; Marchand F.; Balayssac D. Assessment of Mechanical Allodynia in Rats Using the Electronic Von Frey Test. Bio-protocol 2016, 6 (18), e1933 10.21769/BioProtoc.1933.

