# Supplementary material for: Restoring Tactile Sensation Using a Triboelectric Nanogenerator
Source: ACS Nano. 2021 Jun 17;15(7):11087–98. doi: 10.1021/acsnano.0c10141 (PMC8320237; doi:10.1021/acsnano.0c10141)
Supplement: Supplementary file 1 — nn0c10141_si_001.pdf [file nn0c10141_si_001.pdf]

Supporting Information for:
Restoring Tactile Sensation Using a Triboelectric Nanogenerator

Authors: Iftach Shlomy[1]*, Shay Divald[1]*, Keshet Tadmor[2]*, Yael Leichtmann-Bardoogo[1], Amir Arami[3], Ben M. Maoz[1,2,4,§]

[1] Department of Biomedical Engineering, Tel Aviv University, Tel Aviv, 69978, Israel.
[2] Sagol School of Neuroscience, Tel Aviv University, Tel Aviv, 69978, Israel.
[3] Hand Surgery department, Microsurgery and Peripheral nerve surgery unit, Sheba Medical Center, Tel Hashomer, Israel
[4] The Center for Nanoscience and Nanotechnology, Tel Aviv University, Tel Aviv, 69978, Israel.
[§] Corresponding author. Email: bmaoz@tauex.tau.ac.il.
* These authors contributed equally to this work.

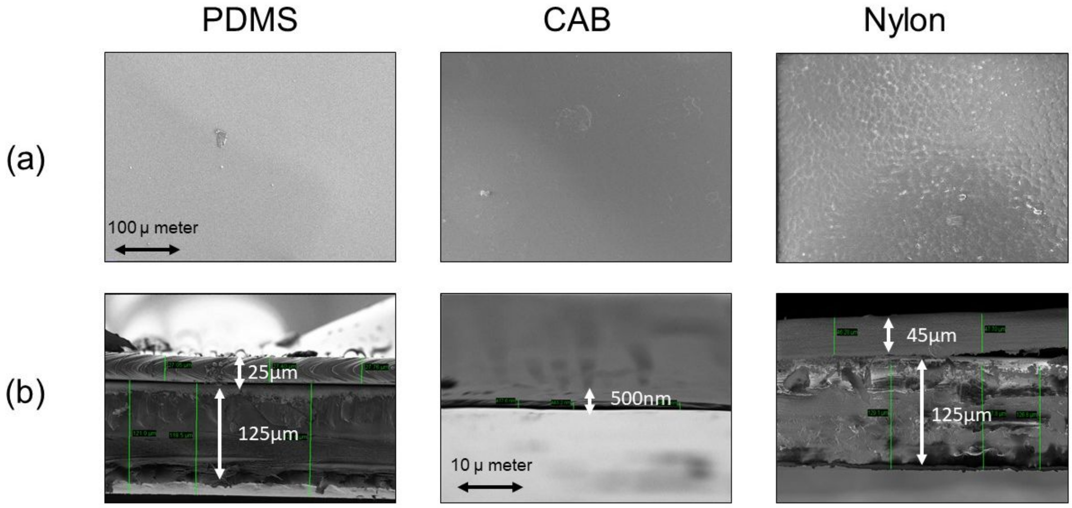

Fig. S1. SEM imaging of TENG-IT dielectric materials. **A.** Top view at ×1000. **B.** Cross-section images at ~×500 used for thickness analysis and conversion. All images were taken with Zeiss© Gemini300 SEM.

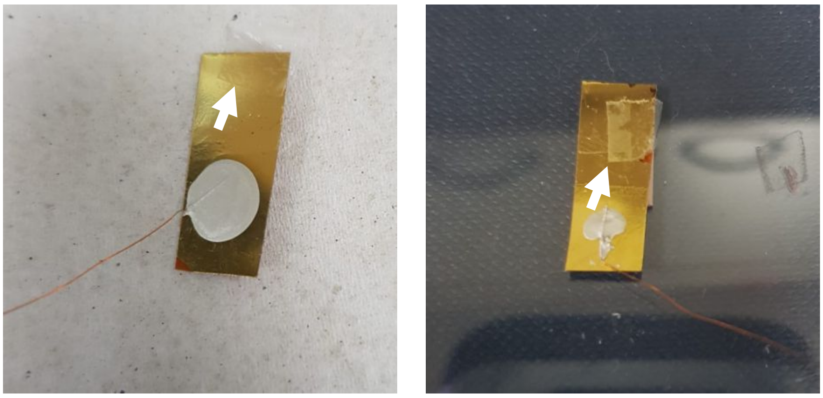

Fig. S2. Nylon (Ny) as a dielectric material. Images of TENG-IT with Ny layer, which has poor adhesion. The gray point is an electrode created with silver paint.

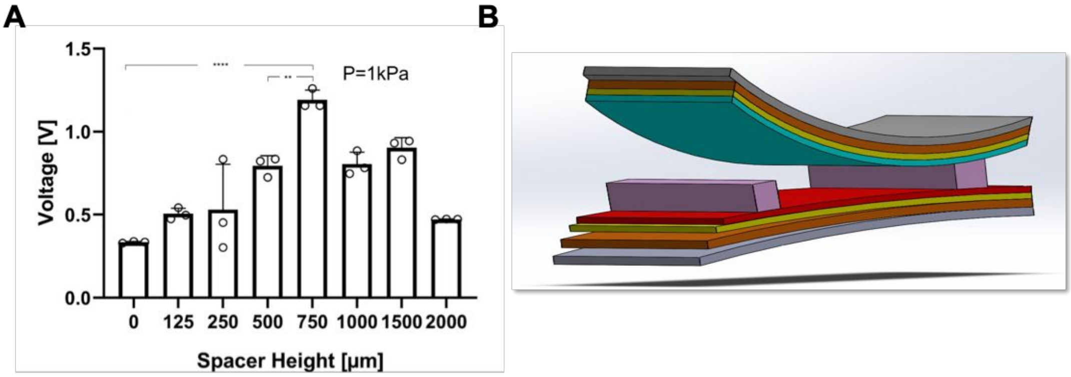

Fig. S3. Spacer optimization. A. Average peak-to-peak output voltage from an encapsulated TENG-IT with different spacer heights, upon application of 1 kPa pressure. N=3 each. B. Schematic of the TENG-IT, according to the following color coding; HVB: gray; Kapton: orange; gold: yellow; PDMS: blue, spacers: Purple, CAB: red.

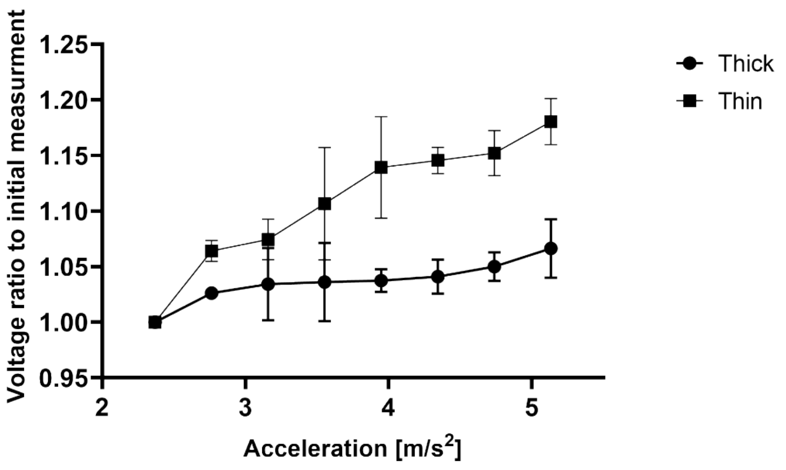

Fig. S4. Kapton thickness affects the TENG-IT's response. Voltage *vs.* pressure for TENG-IT devices (surface area 25 mm^2) made from either a "thick" layer of Kapton (125 μm) or a "thin" layer of Kapton (13 μm).

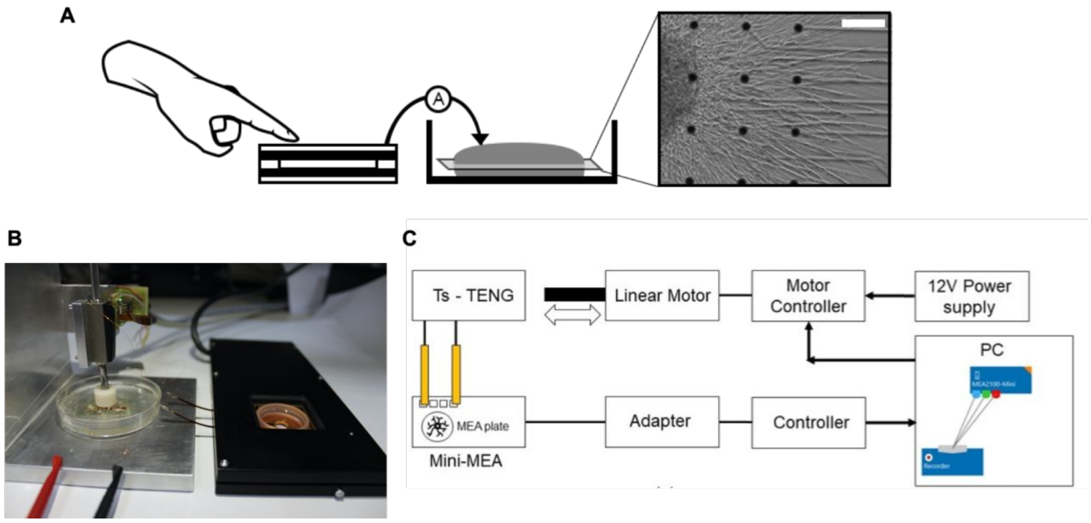

Fig. S5. *In vitro* platform for TENG-IT assessment. A. Schematic of the experimental setup. **B.** Photo of the experimental setup. **C.** Wire configuration of the setup.

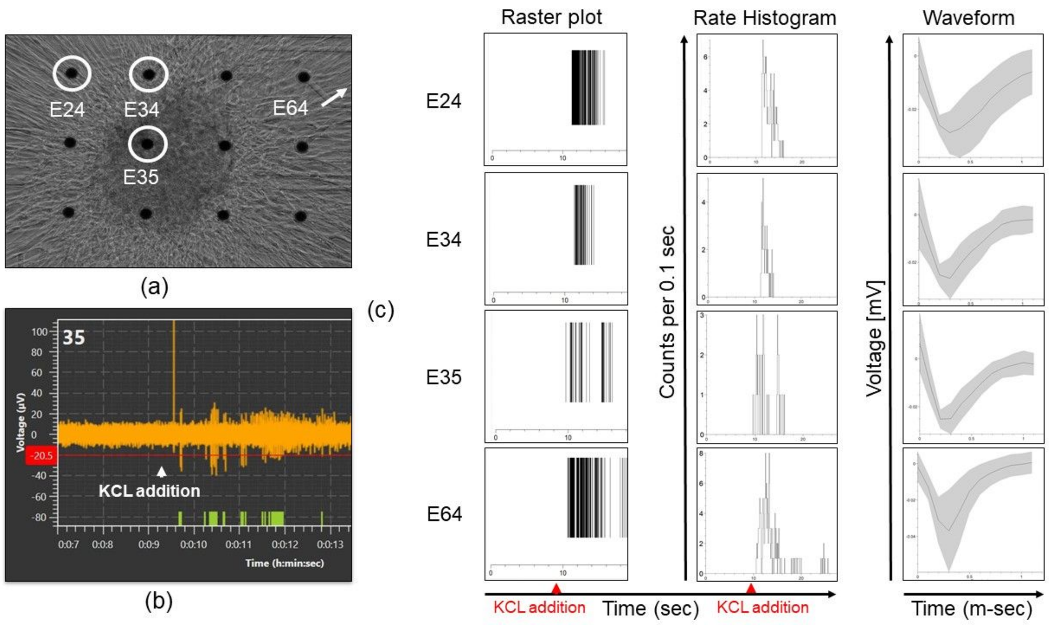

Fig. S6. Validation of model using KCl stimulation. A. ×10 image of DRG on MEA culture at DIV 5, with complete axonal coverage. Analyzed electrodes are marked in white circles. **B.** Raw MEA data, with the threshold for spike definition marked as a horizontal red line, and green vertical lines generating the raster plot of activity. KCl was added to reach 50 mM concentration at t = 10 s. **C.** Data analysis for 4 representative electrodes: Raster plot, rate histogram of spikes per 0.1 seconds, and average waveform shape for each spike (std marked in grey).

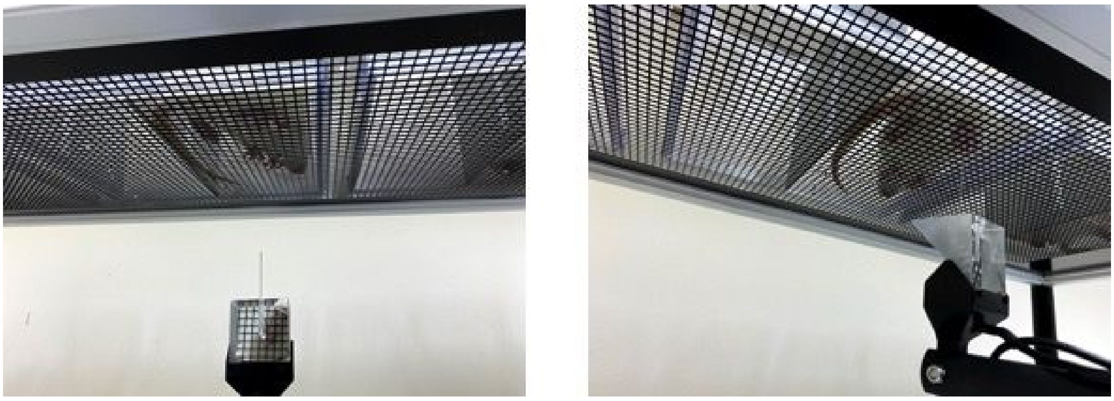

Fig. S7. Setup of the von Frey test. The rat is placed on a wire mesh surface and a pin is pushed into the paw until the rat raises its leg.

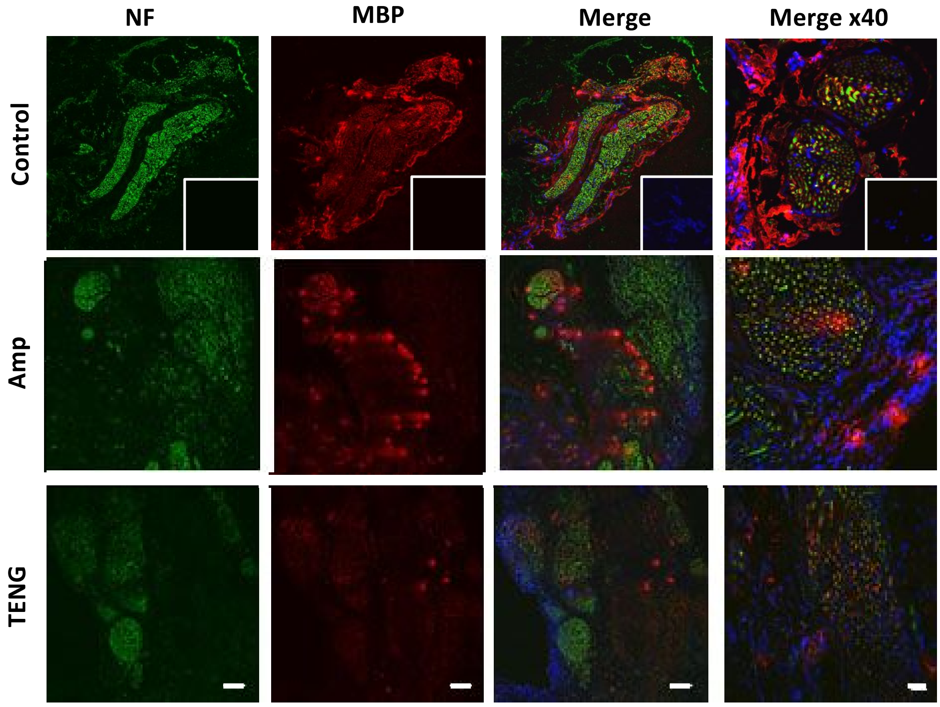

Fig. S8. Immunohistochemistry of the sensory nerve for rats in the control, amputee ("Amp") and TENG-IT ("TENG") groups. To better characterize the nerve response to the transection and device implantation process, the nerve was stained for neurofilaments (NF, green), Myelin (MBP, red), and nucelli (DAPI, blue). Inset presents the negative control; scale bar: 100µm, for ×40: 20µm.

Mov. S1. Triboelectric response of the TENG-IT.
Mov. S2. Typical rat movement after the removal of the sensory nerve.

Table S1

Technology\ parameters	Durability	Detection range	Input power	Size of device	Size of nerve lose that can be repaired	Comments	Refs
**Nerve repair surgeries (grafts)**	>12 months	Up to 5mm two-point discrimination	NA	Up to 3 cm in length	Low success rate when the nerve amputation is more than 5-6cm	Highly dependent on the injury and the regeneration process. ~40% success, when in ~24% of the cases the outcome was reported as "excellent"	[1–4]
**Electronic skin**	4000~ - 14000~ Operation cycles	<18 Pa – 0.7 MPa	External power source	1-8 cm^2	Any gap	Still in research stage and not used in the clinic	[5–10]
**Computer - Brain Interfaces**	2000~ Operation cycles	<22 kPa <1000 kPa	External power source	4 – 16 cm^2	Any gap	High variability between devices	[11–13]
**TENG-IT**	1,000,000 ~ Operation cycles	0-25 kPa	Self-powered	24 mm^2	Any gap		Current study

(1) Braga Silva, J.; Marchese, G. M.; Cauduro, C. G.; Debiasi, M. Nerve Conduits for Treating Peripheral Nerve Injuries: A Systematic Literature Review. *Hand Surg Rehabil* **2017**, *36* (2), 71–85. https://doi.org/10.1016/j.hansur.2016.10.212.

(2) Dunlop, R. L. E.; Wormald, J. C. R.; Jain, A. Outcome of Surgical Repair of Adult Digital Nerve Injury: A Systematic Review. *BMJ Open* **2019**, *9* (3), e025443. https://doi.org/10.1136/bmjopen-2018-025443.

(3) Kornfeld, T.; Vogt, P. M.; Radtke, C. Nerve Grafting for Peripheral Nerve Injuries with Extended Defect Sizes. *Wien Med Wochenschr* **2019**, *169* (9–10), 240–251. https://doi.org/10.1007/s10354-018-0675-6.

(4) Saeki, M.; Tanaka, K.; Imatani, J.; Okamoto, H.; Watanabe, K.; Nakamura, T.; Gotani, H.; Ohi, H.; Nakamura, R.; Hirata, H. Efficacy and Safety of Novel Collagen Conduits Filled with Collagen Filaments to Treat Patients with Peripheral Nerve Injury: A Multicenter, Controlled, Open-Label Clinical Trial. *Injury* **2018**, *49* (4), 766–774. https://doi.org/10.1016/j.injury.2018.03.011.

(5) Chortos, A.; Liu, J.; Bao, Z. Pursuing Prosthetic Electronic Skin. *Nature Materials* **2016**, *15* (9), 937–950. https://doi.org/10.1038/nmat4671.

(6) Li, Z.; Zhu, M.; Shen, J.; Qiu, Q.; Yu, J.; Ding, B. All-Fiber Structured Electronic Skin with High Elasticity and Breathability. *Advanced Functional Materials* **2020**, *30* (6), 1908411. https://doi.org/10.1002/adfm.201908411.

(7) Wang, S.; Ding, L.; Wang, Y.; Gong, X. Multifunctional Triboelectric Nanogenerator towards Impact Energy Harvesting and Safeguards. *Nano Energy* **2019**, *59*, 434–442. https://doi.org/10.1016/j.nanoen.2019.02.060.

(8) Sultana, A.; Ghosh, S. K.; Sencadas, V.; Zheng, T.; Higgins, M. J.; Middya, T. R.; Mandal, D. Human Skin Interactive Self-Powered Wearable Piezoelectric Bio-e-Skin by Electrospun Poly-L-Lactic Acid Nanofibers for Non-Invasive Physiological Signal Monitoring. *J. Mater. Chem. B* **2017**, *5* (35), 7352–7359. https://doi.org/10.1039/C7TB01439B.

(9) Dahiya, A. S.; Morini, F.; Boubenia, S.; Nadaud, K.; Alquier, D.; Poulin-Vittrant, G. Organic/Inorganic Hybrid Stretchable Piezoelectric Nanogenerators for Self-Powered Wearable

Electronics. *Advanced Materials Technologies* **2018**, *3* (2), 1700249. https://doi.org/10.1002/admt.201700249.

(10) Deng, W.; Jin, L.; Zhang, B.; Chen, Y.; Mao, L.; Zhang, H.; Yang, W. A Flexible Field-Limited Ordered ZnO Nanorod-Based Self-Powered Tactile Sensor Array for Electronic Skin. *Nanoscale* **2016**, *8* (36), 16302–16306. https://doi.org/10.1039/C6NR04057H.

(11) Baghaei Naeini, F.; AlAli, A. M.; Al-Husari, R.; Rigi, A.; Al-Sharman, M. K.; Makris, D.; Zweiri, Y. A Novel Dynamic-Vision-Based Approach for Tactile Sensing Applications. *IEEE Transactions on Instrumentation and Measurement* **2020**, *69* (5), 1881–1893. https://doi.org/10.1109/TIM.2019.2919354.

(12) Luo, S.; Zhou, X.; Tang, X.; Li, J.; Wei, D.; Tai, G.; Chen, Z.; Liao, T.; Fu, J.; Wei, D.; Yang, J. Microconformal Electrode-Dielectric Integration for Flexible Ultrasensitive Robotic Tactile Sensing. *Nano Energy* **2021**, *80*, 105580. https://doi.org/10.1016/j.nanoen.2020.105580.

(13) Payeur, P.; Pasca, C.; Cretu, A.-M.; Petriu, E. M. Intelligent Haptic Sensor System for Robotic Manipulation. *IEEE Transactions on Instrumentation and Measurement* **2005**, *54* (4), 1583–1592. https://doi.org/10.1109/TIM.2005.851422.
